# Performance of Multimodal Large Language Models in Detection and Position Assessment of Thoracic Devices on Chest Radiographs

**DOI:** 10.3390/diagnostics16111602

**Published:** 2026-05-23

**Authors:** Hamza Eren Güzel, Cemre Özenbaş, Babak Saravi

**Affiliations:** 1 Department of Radiology, İzmir City Hospital, University of Health Sciences, İzmir 35540, Türkiye; hamzaerenguzel@gmail.com; 2 Department of Radiology, Private Buca Hospital, Tınaztepe University, İzmir 35540, Türkiye; cozenbas@hotmail.com; 3 Department of Oral, Maxillofacial and Facial Plastic Surgery, Medical Faculty and University Hospital Düsseldorf, Heinrich-Heine-University Düsseldorf, 40225 Düsseldorf, Germany

**Keywords:** chest radiography, large language models, thoracic devices, artificial intelligence, device malposition, multimodal AI, diagnostic accuracy, central venous catheter

## Abstract

**Background:** Accurate identification and positioning of thoracic devices on chest radiographs is critical for patient safety in intensive care. Multimodal large language models (LLMs) offer potentially generalizable automated evaluation, but their performance in this domain is underexplored. **Methods:** Three multimodal LLMs (GPT-4o, gpt-4o-2024-08-06; Gemini 3.1 Flash Lite Preview; Claude Sonnet 4.6) were evaluated on 4813 chest radiographs from the RANZCR CLiP dataset for device presence and positioning of ETT, NGT, CVC, and Swan–Ganz catheters. Performance was quantified with 95% Wilson confidence intervals, balanced accuracy, MCC, Cochran’s Q, Bonferroni-corrected McNemar, and Cohen’s/Fleiss’ kappa. Six additional analyses were performed: a blinded paired reader study (n = 377; two board-certified radiologists, blinded to ground truth and to all LLM outputs), external validation on PadChest (n = 200, device-presence detection only—PadChest lacks granular position labels), three-variant prompt-sensitivity analysis (n = 103), repeat-inference stability across three runs (n = 50), systematic error taxonomy, and a failure-case analysis. **Results:** Device-presence performance varied widely across models; abnormal-position sensitivity was uniformly poor (MCC ≤ 0.028; balanced accuracy 0.41–0.53). Inter-model agreement was poor to slight (Fleiss’ κ: 0.005–0.383 for presence; −0.280 to −0.025 for classification). Radiologists numerically outperformed all three LLMs in 42/42 paired comparisons; the superiority was statistically significant after Bonferroni correction in 33/42 (32/42 at *p* < 0.001). PadChest replicated the negative finding for device-presence detection (malposition not externally validated). Prompts and inference stochasticity introduced 2–3× sensitivity swings and run-to-run κ from 0.20 to 0.85. Case failures concentrated systematically in multi-device cases (*p* < 0.0001) but not in abnormal-position cases (*p* = 0.14). **Conclusions:** Current general-purpose multimodal LLMs are not yet reliable for autonomous thoracic-device assessment; their failure patterns are structurally characterizable across models, prompts, and case types and support, at most a circumscribed role, as adjunct device-presence screening tools. The findings do not generalize to purpose-built, regulator-approved clinical AI systems.

## 1. Introduction

Chest radiography remains one of the most commonly performed imaging modalities in clinical practice, particularly in emergency and intensive care settings where rapid decision-making is critical. Accurate identification and positioning of thoracic devices—such as endotracheal tubes (ETT), nasogastric tubes (NGT), and central venous catheters (CVC)—are essential to ensure patient safety and to avoid potentially life-threatening complications associated with malpositioned devices [1,2]. Despite its routine use, interpretation of chest radiographs in critically ill patients is often challenging due to suboptimal image quality, patient positioning, overlapping devices, and complex clinical contexts [3].

In recent years, artificial intelligence (AI), particularly deep learning-based computer vision models, has demonstrated promising performance in various radiologic tasks, including abnormality detection and device localization on chest radiographs [4,5]. AI-based approaches have also been explored for radiology workflow optimization, including automated classification of radiology reports and triage systems, demonstrating high diagnostic performance and potential clinical utility [6]. Recent work on deep learning for tube and line detection in critical care settings has shown that dedicated models can achieve performance comparable to radiology residents, highlighting both the promise and current ceiling of supervised approaches [7]. However, these models are typically task-specific, require extensive labeled datasets for training, and often lack flexibility in adapting to new clinical questions without retraining [8].

More recently, the emergence of large language models (LLMs), especially multimodal variants capable of processing both text and images, has introduced a new paradigm in medical image interpretation. These models, including GPT-based systems, Gemini, and Claude, have shown the ability to perform complex reasoning tasks and generate structured outputs from imaging data without explicit task-specific training [9]. Early studies suggest that multimodal LLMs may achieve clinically meaningful performance across a range of radiologic applications, raising the possibility of more generalizable and scalable AI solutions [10,11]. A recent evaluation of GPT-4V on chest radiograph interpretation demonstrated moderate accuracy in detecting radiologic findings while highlighting persistent limitations in spatial reasoning and subtle finding detection [12]. Similarly, comparative studies of GPT-4o, Claude 3 Opus, and Gemini Pro in diagnostic radiology cases have revealed substantial inter-model variability, with no single model consistently outperforming others across all tasks [13,14].

A recent umbrella review additionally confirmed the strong diagnostic performance of dedicated AI models when used as second readers in radiology workflows [15], and a multi-case multi-reader study demonstrated that AI-assisted interpretation improved radiologist accuracy for identifying malpositioned endotracheal tubes from 79.3% to 89.0% [16], establishing meaningful benchmarks for task-specific systems against which generalist multimodal LLMs may be compared.

Nevertheless, the application of LLMs to chest radiography, particularly for device detection and positional assessment, remains underexplored. Device evaluation presents unique challenges, including the need for precise spatial reasoning, differentiation between acceptable and unsafe positioning, and handling of multiple coexisting devices within a single image [3]. Furthermore, unlike traditional AI models, LLMs rely heavily on prompt design and implicit reasoning, which may influence performance variability across different tasks and device types [9,11].

A growing body of literature has emphasized the potential of multimodal foundation models to transform radiology workflows while also underscoring the need for rigorous benchmarking against established standards [17,18]. However, comparative evaluations of different state-of-the-art LLMs in realistic, heterogeneous chest radiography datasets remain limited, and systematic assessment of inter-model agreement has been largely absent from the literature.

Therefore, the aim of this study was to systematically evaluate and compare the performance of three contemporary multimodal LLMs—GPT-4o, Gemini 3.1 Flash Lite Preview, and Claude Sonnet 4.6—in detecting and classifying thoracic devices on chest radiographs. Using a large, real-world dataset with expert annotations, we assessed both device presence detection and the ability to distinguish between normal and abnormal positioning, and additionally, we evaluated inter-model agreement to determine the consistency and reliability of LLM-based assessments in this clinically relevant task. To position the work as a structured safety benchmark for current general-purpose multimodal LLMs in this clinical task, the study additionally provides (i) a paired reader study with two board-certified radiologists serving as a human comparator, (ii) external validation on the independent PadChest dataset, (iii) prompt-sensitivity analysis across three prompt variants, (iv) repeat-inference stability assessment, and (v) systematic error taxonomy. We acknowledge that documenting suboptimal general-purpose LLM performance on a narrow radiological task is largely an expected finding in this study; the novel contribution of this work lies not in demonstrating that LLMs fail, but in systematically characterizing *where* and *how* this failure occurs: across three specific models, four device types, three prompt variants, multiple inference runs, and a structured error taxonomy, with paired comparison against blinded radiologists on the same images and an external dataset (for device presence) demonstrating that the principal findings are not RANZCR-specific.

## 2. Materials and Methods

### 2.1. Dataset

This study utilized the RANZCR CLiP Kaggle Dataset, a publicly available chest radiography dataset for the detection and classification of medical devices on chest X-rays [19]. The dataset comprises approximately 40,000 radiographic examinations annotated for the presence and positioning of critical devices, including endotracheal tubes (ETT), nasogastric tubes (NGT), central venous catheters (CVC), and Swan–Ganz catheters.

Images in the dataset are provided in JPEG format with varying resolutions and acquisition parameters, reflecting real-world heterogeneity in radiographic imaging. The dataset includes both portable and standard posterior–anterior/anterior–posterior chest radiographs, encompassing a wide range of patient positioning, exposure conditions, and device configurations. Ground truth annotations were established based on expert radiologist labeling.

For the purposes of this study, a subset of 5000 cases was randomly sampled from the full dataset to ensure computational feasibility while maintaining diversity in device types and positioning scenarios. Sampling was performed using simple random sampling without replacement (Python NumPy random.choice with fixed seed for reproducibility) on the full RANZCR CLiP train set. The full sampled list of 5000 StudyInstanceUIDs is provided as Supplementary Material. The study was conducted in accordance with the Declaration of Helsinki and approved by the Non-Interventional Research Ethics Committee of İzmir Tınaztepe University, İzmir, Türkiye (approval no. MAEK2025/114; approval date: 2 December 2025).

### 2.2. Characteristics

Images were provided in JPEG format and consisted of standard chest radiographs acquired under routine clinical conditions. The majority of images were portable anterior–posterior (AP) projections, frequently obtained in intensive care or emergency settings. Image resolution and compression levels varied across cases, reflecting real-world heterogeneity.

Radiographs were grayscale with typical 8-bit depth and demonstrated variability in exposure, contrast, and noise levels. Patient positioning was often suboptimal, with mild rotation and variable inspiration levels commonly observed. External devices and lines were frequently present, sometimes overlapping with anatomical structures, increasing interpretation complexity.

The dataset included images with multiple simultaneous devices, diverse anatomical coverage, and varying degrees of image quality, thereby providing a realistic representation of daily clinical practice. Images were submitted to each model in their native JPEG form without preprocessing; no resizing, compression, or normalization was applied, preserving the diagnostic image properties as encountered in real-world clinical settings.

### 2.3. Study Design and Classification Scheme

The study was designed to evaluate the performance of large language models (LLMs) in detecting and classifying the presence and positioning of thoracic devices on chest radiographs.

Devices evaluated included endotracheal tube (ETT), nasogastric tube (NGT), central venous catheter (CVC), and Swan–Ganz catheter.

Device positioning was simplified into a binary classification framework: Normal (correctly positioned or clinically acceptable) and Abnormal (clearly malpositioned or unsafe). Importantly, slightly misplaced but clinically acceptable devices were categorized as normal, in order to reflect real-world clinical tolerance and reduce ambiguity in borderline cases.

Each device was evaluated independently. For ETT, NGT, and CVC, only one of the two categories (normal or abnormal) could be assigned per case. The Swan–Ganz catheter was assessed as a binary variable (present or absent).

It should be noted that the number of abnormal positioning cases was very low for certain devices, particularly ETT (10 abnormal out of 1276 device-present cases, 0.8%) and NGT (38 out of 1251, 3.0%). This extreme class imbalance limits the statistical power and reliability of sensitivity estimates for abnormal detection, and classification results for ETT should be interpreted with particular caution.

### 2.4. Model Selection and Implementation

Three state-of-the-art multimodal large language models were evaluated via their respective APIs [20]: GPT-4o (OpenAI), Gemini 3.1 Flash Lite Preview (Google), and Claude Sonnet 4.6 (Anthropic). All models were accessed between 15 March and 31 March 2026. The exact API model identifiers were as follows: gpt-4o-2024-08-06 for GPT-4o (model snapshot recorded in the API logs); gemini-3.1-flash-lite-preview, resolved by the API on the access date (15 March 2026) to the then-current preview model; and claude-sonnet-4-6, resolved by the API on the access date (31 March 2026) to the then-current Sonnet 4.6 release. The two non-snapshot aliases (Gemini and Claude) provide model identity but not an immutable point-in-time hash; the access dates above are therefore the relevant reproducibility anchors for these two models.

All models were accessed through their official API interfaces and evaluated under comparable conditions. Default inference settings were used without additional fine-tuning. Because each provider has slightly different default sampling temperatures, top-p, top-k, and penalty settings, inference conditions were not fully standardized across models. This is a known limitation of multi-vendor LLM benchmarking and is addressed quantitatively by the repeat-inference stability analysis (Section 2.10), which characterizes the resulting intra-model variability. Each image was independently processed by each model using a standardized prompt to ensure consistency across evaluations. For full reproducibility, max_tokens was set to 500 for all three models. Sampling temperature was not explicitly set and used each provider’s API default. Top-p, top-k, and other generation hyperparameters were left at provider defaults. The GPT-4o request additionally included a system message instructing the model to support a de-identified radiology research study, classify devices according to the user prompt, and avoid refusals or disclaimers; no system prompt was used for Gemini or Claude. Images were transmitted as base64-encoded JPEG data with detail set to “high” for GPT-4o and provider-default detail for the other two models. Approximate API costs across all 5000 calls per model were ~USD 25–40 for GPT-4o, ~USD 30–45 for Claude Sonnet 4.6, and ~USD 5–10 for Gemini 3.1 Flash Lite Preview.

Of the 5000 cases, 20 (GPT-4o), 47 (Gemini), and 122 (Claude) were not successfully processed; these model-specific failure counts sum to 189, but two cases overlapped between models (failed in more than one model), so the union of unique unprocessed cases was 187, leaving 4813 cases successfully processed by all three models. The predominant failure mode for Claude was a content-policy refusal of the form “I can’t review this image, because it is a medical file,” arising from Claude’s safety classifier; this refusal was absent for GPT-4o because the system prompt explicitly instructed the model not to refuse and absent for Gemini due to its less strict default safety policies. Failures for GPT-4o and Gemini were primarily empty responses or JSON parsing errors.

### 2.5. Prompt Design

A standardized prompt was used for all models to ensure consistent and reproducible evaluation. The prompt defined the target devices, classification criteria, and output structure and was provided identically to each model as follows:

“Analyze the provided chest X-ray and classify the presence and position of the following devices.

DEFINITIONS:

ETT: Endotracheal Tube

NGT: Nasogastric Tube

CVC: Central Venous Catheter

Swan-Ganz Catheter: Pulmonary artery catheter

CLASSIFICATION RULES:

For each device:

“Normal” = correctly positioned or acceptable

“Abnormal” = clearly malpositioned or unsafe

GENERAL RULES:

If a device is NOT present → all its categories must be 0

If a device is visible, you MUST classify it and assign exactly one category as 1

Search carefully for each device

For each device (ETT, NGT, CVC), ONLY ONE of [Abnormal, Normal] can be 1

Devices are independent; multiple devices can be present and have 1 simultaneously

Swan-Ganz is binary: 1 if present, 0 if absent

OUTPUT FORMAT:

Return ONLY valid JSON. Do not include any text before or after the JSON.

{ “ETT—Abnormal”: 0, “ETT—Normal”: 0, “NGT—Abnormal”: 0, “NGT—Normal”: 0, “CVC—Abnormal”: 0, “CVC—Normal”: 0, “Swan Ganz Catheter Present”: 0 }”

Prompt selection was preceded by an exploratory phase in which several alternative prompt formulations were tested on a 200-image preliminary subset across multiple candidate models, before selecting the prompt above and the three models reported. The 200 cases used during this exploratory phase were drawn from the same RANZCR CLiP train set but did not overlap with the 4813-case final analysis cohort (the two samples were drawn independently before cohort finalization). Consequently, prompt selection was performed entirely out-of-sample with respect to the analysis cohort, eliminating direct data leakage from prompt-tuning. Given the well-documented prompt-sensitivity of multimodal LLMs, a formal three-variant prompt sensitivity analysis was conducted to quantify this effect (Section 2.9).

Two minor model-specific adaptations were applied to the shared core prompt and are disclosed for transparency: the Claude prompt added an explicit tie-breaker rule (“If uncertain between normal and abnormal → classify as abnormal”), and the GPT-4o and Gemini prompts added a clarifying rule that “devices are independent.” These adaptations are unlikely to drive the principal findings, since (a) Claude’s tie-breaker rule did not raise its abnormal sensitivity (which remained near zero across all device types), and (b) the device-independence statement was already implicit in the JSON output schema common to all three models.

### 2.6. Statistical Analysis

All statistical analyses were performed using Python (version 3.12) with the scikit-learn (version 1.8), SciPy (version 1.17), and statsmodels (version 0.14) libraries. For device presence detection and normal versus abnormal classification, accuracy, sensitivity, specificity, precision, and F1 score were computed for each model–device combination. Given the substantial class imbalance—particularly for abnormal positioning (ETT: 10/1276, NGT: 38/1251, CVC: 347/4707)—balanced accuracy and the Matthews correlation coefficient (MCC) were additionally reported as imbalance-robust metrics [21]. All performance metrics were accompanied by 95% Wilson score confidence intervals.

The pre-specified null hypothesis (H0) for malposition detection was that LLM performance does not exceed chance (MCC = 0; balanced accuracy = 0.5). The pre-specified H0 for inter-model agreement was Fleiss’ κ = 0 (no above-chance agreement). For pairwise model comparisons, the McNemar test was used with exact binomial computation. To account for simultaneous three-way model comparison, Cochran’s Q test was applied as an omnibus test, followed by post hoc pairwise McNemar tests with Bonferroni correction for multiple comparisons (12 tests for device presence, 9 tests for classification). A corrected *p*-value < 0.05 was considered statistically significant.

Inter-model agreement was evaluated using Cohen’s kappa for pairwise comparisons and Fleiss’ kappa for overall three-model agreement, computed separately for device presence detection and normal versus abnormal classification. Kappa values were interpreted according to Landis and Koch: <0 poor, 0–0.20 slight, 0.21–0.40 fair, 0.41–0.60 moderate, 0.61–0.80 substantial, and 0.81–1.00 almost perfect [22]. Confusion matrices were generated for each model–device pair and are provided as Supplementary Material.

Subgroup analyses were performed by stratifying cases according to the number of concurrent devices present (0, 1, 2, or 3+ devices) to evaluate whether multi-device complexity affected model performance. This study adhered to the Standards for Reporting Diagnostic Accuracy Studies (STARD) 2015 guidelines, extended for the present AI-centric diagnostic accuracy study by the emerging STARD-AI reporting items [23,24]; thecompleted STARD-AI checklist (which extends STARD 2015 with AI-extension items) is provided as Supplementary Table S3.

### 2.7. Blinded Radiologist Reader Study

To benchmark LLM performance against expert human reads on the same images, two board-certified/board-eligible radiologists (co-authors H.E.G. and C.Ö.) independently re-read a stratified-enriched subset of 377 chest radiographs drawn from the 4813-case cohort. Both readers were blinded to ground truth and to all LLM outputs. Enrichment was applied because abnormal-position prevalence in the full cohort was too low to support meaningful paired statistical comparisons (ETT 0.8%, NGT 3.0%). The enriched subset comprised all 10 ETT-abnormal cases, all 38 NGT-abnormal cases, 80 CVC-abnormal cases (40 with concurrent devices, 40 with CVC only), 60 ETT-normal controls, 100 NGT-normal controls, 40 Swan–Ganz-positive cases, and 50 CVC-only-normal controls. Cases were randomly drawn from each stratum with a fixed seed for reproducibility. After accounting for inter-stratum overlap, 377 unique radiographs were assessed.

Both readers were blinded to (i) the gold-standard RANZCR labels, (ii) all three LLM outputs, and (iii) the co-reader’s assessments until both submitted their forms. Cases were presented to each reader in independently randomized order. Image quality was rated on a three-point scale (adequate/suboptimal/non-diagnostic). For each case, the readers recorded device presence (binary) and, if present, position (normal/abnormal) for ETT, NGT, and CVC, plus binary Swan–Ganz presence. The reader study was performed independently by the two radiologists on Windows OS computers using the built-in Microsoft Photos application; because images were already provided as JPEGs (not DICOM), no dedicated DICOM viewer was used. Both readers used 15.6-inch laptop screens at Full HD resolution (1920 × 1080 pixels). No zoom, window/level adjustment, or other post-processing tools were applied. Both readers were certified by the European Board of Radiology (EDiR) and Turkish board certified. We deliberately matched the readers’ viewing conditions to those received by the LLMs (identical JPEG inputs at the same nominal resolution, with no DICOM-viewer assistance, window/level adjustment, or zoom) in order to compare LLM performance against expert reads at equivalent input fidelity. We acknowledge that this is not how radiologists typically read in clinical practice (where DICOM viewers, windowing, magnification, and multi-monitor diagnostic workstations are standard); reader performance under standard clinical viewing conditions would likely be higher than reported here, which would only further widen the observed gap between human and LLM performance. We note that these viewing conditions are comparable to, rather than substantially more favorable than, the LLMs—both received the same JPEGs at the same nominal resolution—but a fully matched condition (e.g., reader viewing the API-encoded JPEG byte-stream) was not enforced and is acknowledged as a limitation.

Inter-reader agreement was quantified by Cohen’s kappa for each device task. Reader vs. LLM paired comparisons used the McNemar test with Bonferroni correction over the 42 paired comparisons (2 readers × 3 LLMs × 7 device–task pairs). Reader-vs-gold-standard performance was reported with 95% Wilson score confidence intervals on the same metrics as the main analysis.

This blinded radiologist study should not be interpreted as a fully independent multi-center reader study: both readers are co-authors of this manuscript, the cohort was a stratified-enriched subset rather than a consecutive series, and the study was single-center. The comparison is best characterized as an internal benchmark of LLM performance against expert human reads on the same images. The risk of optimism bias arising from the co-author status of the readers is mitigated by their being fully blinded to ground-truth labels and to all LLM outputs at the time of reading but cannot be eliminated and is acknowledged as a limitation.

### 2.8. External Validation on PadChest

To address generalizability, an external validation was conducted on the PadChest dataset (Bustos et al., Spain) [25]. PadChest provides device-type labels (endotracheal tube, NSG tube, central venous catheter, tracheostomy, pacemaker), allowing for presence-detection validation across an independent population. PadChest does not provide granular position labels comparable to RANZCR CLiP, so the external validation was limited to device presence detection.

From the 110,633 PadChest images with valid PA/AP projection metadata, a stratified subset of 500 cases was sampled (125 ETT-positive, 125 NGT-positive, 125 CVC-positive, 125 device-negative). Of these, 205 images were successfully extracted for analysis through the BIMCV public-access tier; the remaining 295 cases could not be obtained within the practical access constraints of that public tier. After alignment with all three LLM outputs (GPT-4o had 5 JSON-parsing failures), 200 cases were available for the final external-validation analysis. PadChest images are 16-bit grayscale PNGs that were normalized to 8-bit JPEG (0.5–99.5 percentile windowing, max long edge 1568 px, JPEG quality 92) before submission to the LLM APIs to ensure compatibility with the multimodal vision encoders while preserving diagnostic contrast. All three LLMs were re-run on the 205 PadChest images using the same prompt and inference parameters as in the main analysis. Performance metrics for ETT, NGT, and CVC presence detection were computed and compared head-to-head with the corresponding RANZCR results. Inter-model agreement on PadChest was also computed (Cohen’s and Fleiss’ kappa).

### 2.9. Prompt Sensitivity Analysis

To formally quantify LLM sensitivity to prompt phrasing, a stratified subset of 103 RANZCR cases (stratified to include all major device-stratum combinations) was re-evaluated by all three models using two additional prompt variants alongside the baseline:

- V1 (baseline): the prompt used in the main study (Section 2.5).
- V2 (chain-of-thought): identical task definition with an explicit pre-output reasoning sequence (“Before producing the JSON, reason step by step about each device: ETT trachea/carina relationship, NGT esophageal path, CVC route and tip location, Swan–Ganz pulmonary artery route”). The model was instructed to perform reasoning internally and produce only valid JSON in the output.
- V3 (minimal/concise): a brief one-paragraph prompt asking the model to identify the four devices and return JSON in the same schema, with no rules elaboration.

All three models were run on the same 103 cases for each variant. Performance metrics (sensitivity, specificity, MCC, balanced accuracy) were compared across variants. The full text of all three prompt variants is provided as Supplementary Methods S1.

### 2.10. Repeat-Inference Stability Analysis

To assess intra-model stochasticity—relevant given that the main study used each provider’s default sampling temperature—a stratified subset of 50 RANZCR cases was re-evaluated by each model on three independent runs using the V1 baseline prompt. The three runs included the original main-study run plus two re-runs performed within a single calendar day. Pairwise Cohen’s kappa was computed across runs for each model–device combination, and the mean of the three pairwise kappas reported as a stability score.

### 2.11. Error Taxonomy

To characterize qualitative failure modes, a systematic error analysis was conducted across the full 4813-case cohort. Five error categories were enumerated: (i) ETT/NGT confusion (a gold-standard ETT-only case classified as NGT-only, or vice versa), (ii) device hallucination (model predicting any device on gold-standard device-absent cases), (iii) rare device misses (Swan–Ganz misses), (iv) abnormal-position misses (gold abnormal, model normal-or-absent), and (v) multi-device degradation (per-device accuracy in single-device vs. multi-device cases). Rates were reported separately for each model.

### 2.12. Failure-Case Analysis

To assess whether processing failures were randomly distributed or systematically concentrated in clinically more complex cases (and thus biased performance estimates), we tested associations between case failure and gold-standard features: number of concurrent devices, presence of each device type (ETT, NGT, CVC, Swan–Ganz), and presence of any abnormal positioning. Chi-square test for trend was used for the device-count analysis; Fisher exact tests were used for individual binary predictors, with odds ratios and 95% confidence intervals.

## 3. Results

**Figure 1:**
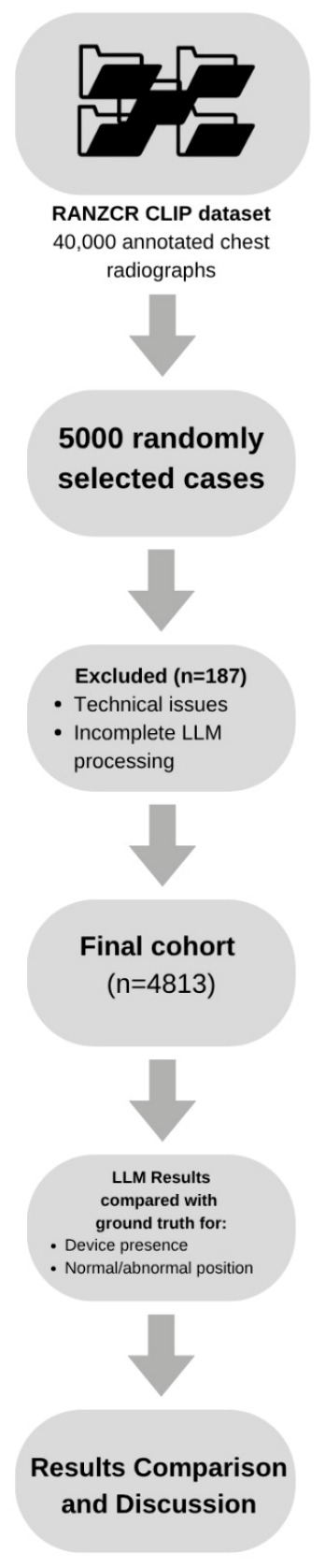
A flowchart illustrating case selection from the RANZCR CLiP dataset, random sampling of the study subset, exclusion of cases due to technical processing issues across models, and the final study population included in the analysis.

A total of 5000 cases were initially included in the study. Due to technical processing limitations, 20 cases in GPT, 47 cases in Gemini, and 122 cases in Claude could not be processed. After excluding overlapping unprocessed cases, a total of 4813 successfully processed cases were included in the final analysis. The study selection process is summarized in Figure 1.

In the reference standard, device presence was identified in 1276 cases for endotracheal tubes (ETT), 1251 cases for nasogastric tubes (NGT), 4707 cases for central venous catheters (CVC), and 120 cases for Swan–Ganz catheters. Among device-present cases, abnormal positioning was observed in 10 ETT cases (0.8%), 38 NGT cases (3.0%), and 347 CVC cases (7.4%).

### 3.1. Device Presence Detection

The performance of the models in detecting device presence is summarized in Table 1. Accuracy ranged from 0.465 (95% CI: 0.451–0.479) for Claude CVC to 0.969 (95% CI: 0.964–0.974) for Gemini Swan–Ganz. Balanced accuracy ranged from 0.376 to 0.900, with MCC values ranging from −0.073 to 0.754, reflecting substantial variability in model performance across device types.

Gemini achieved the highest sensitivity for ETT detection (0.912; 95% CI: 0.895–0.927) and CVC detection (0.850; 95% CI: 0.839–0.860). Claude demonstrated the highest specificity for ETT (0.912; 95% CI: 0.903–0.921) and NGT (0.855; 95% CI: 0.843–0.866). CVC detection showed high sensitivity but markedly low specificity across all models (range: 0.085–0.283), indicating systematic overcalling. Swan–Ganz catheter detection was characterized by very low sensitivity (range: 0.017–0.242) despite high specificity (range: 0.895–0.992).

**Table 1:** Device presence detection performance with 95% confidence intervals.

Model	Device	Accuracy (95% CI)	Sensitivity (95% CI)	Specificity (95% CI)	Precision (95% CI)	F1 (95% CI)	Bal. Acc.	MCC
GPT	ETT	0.746 (0.733–0.758)	0.534 (0.506–0.561)	0.822 (0.810–0.835)	0.520 (0.493–0.547)	0.527 (0.513–0.541)	0.678	0.353
GPT	NGT	0.706 (0.693–0.719)	0.395 (0.368–0.422)	0.816 (0.803–0.828)	0.430 (0.401–0.458)	0.411 (0.398–0.425)	0.605	0.217
GPT	CVC	0.621 (0.607–0.634)	0.631 (0.617–0.644)	0.170 (0.110–0.253)	0.971 (0.965–0.977)	0.765 (0.753–0.777)	0.400	−0.061
GPT	Swan	0.879 (0.869–0.888)	0.242 (0.174–0.326)	0.895 (0.886–0.904)	0.056 (0.039–0.079)	0.090 (0.083–0.099)	0.568	0.069
Gemini	ETT	0.894 (0.885–0.902)	0.912 (0.895–0.927)	0.887 (0.877–0.897)	0.745 (0.723–0.766)	0.820 (0.809–0.831)	0.900	0.754
Gemini	NGT	0.727 (0.715–0.740)	0.441 (0.414–0.469)	0.828 (0.815–0.840)	0.474 (0.445–0.503)	0.457 (0.443–0.471)	0.635	0.276
Gemini	CVC	0.833 (0.822–0.843)	0.850 (0.839–0.860)	0.085 (0.045–0.154)	0.976 (0.971–0.981)	0.909 (0.900–0.916)	0.467	−0.027
Gemini	Swan	0.969 (0.964–0.974)	0.083 (0.046–0.147)	0.992 (0.989–0.994)	0.204 (0.115–0.336)	0.118 (0.110–0.128)	0.538	0.117
Claude	ETT	0.825 (0.814–0.836)	0.583 (0.556–0.610)	0.912 (0.903–0.921)	0.706 (0.678–0.733)	0.639 (0.625–0.652)	0.748	0.529
Claude	NGT	0.781 (0.769–0.792)	0.568 (0.541–0.596)	0.855 (0.843–0.866)	0.579 (0.552–0.607)	0.574 (0.560–0.588)	0.712	0.426
Claude	CVC	0.465 (0.451–0.479)	0.469 (0.455–0.483)	0.283 (0.206–0.375)	0.967 (0.959–0.973)	0.631 (0.618–0.645)	0.376	−0.073
Claude	Swan	0.965 (0.959–0.970)	0.017 (0.005–0.059)	0.989 (0.985–0.992)	0.037 (0.010–0.125)	0.023 (0.019–0.028)	0.503	0.008

Bal. Acc. = balanced accuracy; MCC = Matthews correlation coefficient; CI = confidence interval. Cochran’s Q test demonstrated statistically significant overall differences among the three models for all device types (all *p* < 0.001). Pairwise comparisons with Bonferroni correction are presented in Table 2. After correction for multiple comparisons, the comparison between GPT and Gemini for NGT presence detection did not reach statistical significance (*p*_corrected = 0.113), and the comparison between Gemini and Claude for Swan–Ganz detection was also non-significant (*p*_corrected = 0.475). All other pairwise comparisons remained statistically significant (*p*_corrected < 0.05).

**Table 2:** Cochran’s Q test and pairwise McNemar tests with Bonferroni correction for device presence detection.

Device	Cochran’s Q	Cochran *p*	Comparison	*p* (Raw)	*p* (Corrected)	Better
ETT	433.2	<0.001	GPT vs. Gemini	<0.001	<0.001	Gemini
ETT	433.2	<0.001	GPT vs. Claude	<0.001	<0.001	Claude
ETT	433.2	<0.001	Gemini vs. Claude	<0.001	<0.001	Gemini
NGT	97.1	<0.001	GPT vs. Gemini	0.009	0.113	Gemini
NGT	97.1	<0.001	GPT vs. Claude	<0.001	<0.001	Claude
NGT	97.1	<0.001	Gemini vs. Claude	<0.001	<0.001	Claude
CVC	1883.0	<0.001	GPT vs. Gemini	<0.001	<0.001	Gemini
CVC	1883.0	<0.001	GPT vs. Claude	<0.001	<0.001	GPT
CVC	1883.0	<0.001	Gemini vs. Claude	<0.001	<0.001	Gemini
Swan	605.0	<0.001	GPT vs. Gemini	<0.001	<0.001	Gemini
Swan	605.0	<0.001	GPT vs. Claude	<0.001	<0.001	Claude
Swan	605.0	<0.001	Gemini vs. Claude	0.040	0.475	Gemini

**Figure 2:**
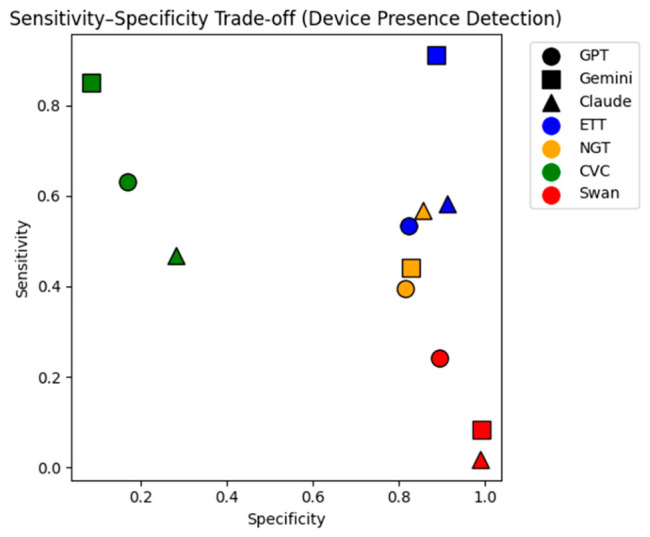
Sensitivity–specificity trade-off for device presence detection across models and device types. Each point represents a device–model pair. Marker shapes indicate models (circle: GPT, square: Gemini, triangle: Claude), while colors represent device types (ETT, NGT, CVC, Swan–Ganz).

The sensitivity–specificity trade-off for device presence detection is illustrated in Figure 2.

### 3.2. Normal vs. Abnormal Classification

Among cases with confirmed device presence, the performance of the models in distinguishing normal from abnormal positioning is summarized in Table 3. All models showed consistently poor sensitivity for detecting abnormal positioning across all device types. For ETT, sensitivity for abnormal detection ranged from 0.000 (Claude; 95% CI: 0.000–0.278) to 0.600 (Gemini; 95% CI: 0.313–0.832), though the latter should be interpreted cautiously given only 10 abnormal ETT cases. For NGT, abnormal sensitivity ranged from 0.000 (Claude) to 0.184 (GPT; 95% CI: 0.092–0.334). For CVC, abnormal sensitivity ranged from 0.020 (Claude; 95% CI: 0.010–0.041) to 0.291 (Gemini; 95% CI: 0.246–0.341).

Balanced accuracy was near chance level (0.50) for all model–device combinations, ranging from 0.411 to 0.527. MCC values were uniformly near zero (range: −0.038 to 0.028), confirming that no model achieved meaningful discrimination between normal and abnormal positioning.

Notably, Claude was identified as the statistically “better” model in the majority of pairwise classification comparisons (Table 4). However, this finding reflects Claude’s strong normality bias—achieving high overall accuracy by classifying nearly all cases as normal—rather than superior diagnostic performance. As shown in Table 3, Claude’s sensitivity for abnormal positioning was near zero for all device types (MCC ≤ 0.008), and its balanced accuracy did not exceed 0.502. Thus, the McNemar test results for classification should be interpreted in the context of the MCC and balanced accuracy metrics, which more accurately capture clinically meaningful discrimination.

### 3.3. Inter-Model Agreement

Inter-model agreement statistics are presented in Table 5. For device presence detection, Cohen’s kappa values ranged from −0.001 (GPT vs. Gemini for Swan–Ganz) to 0.501 (Gemini vs. Claude for ETT), corresponding to poor-to-moderate agreement. Fleiss’ kappa for three-model agreement ranged from 0.005 (Swan–Ganz, indicating virtually no agreement) to 0.383 (ETT, indicating fair agreement).

For normal versus abnormal classification, inter-model agreement was uniformly poor. All pairwise Cohen’s kappa values were near zero (range: −0.045 to 0.014), and Fleiss’ kappa values were negative for all device types (range: −0.280 to −0.025), indicating agreement worse than chance. This finding suggests that the three models employ fundamentally different internal decision strategies when assessing device positioning.

### 3.4. Subgroup Analysis by Device Complexity

**Figure 3:**
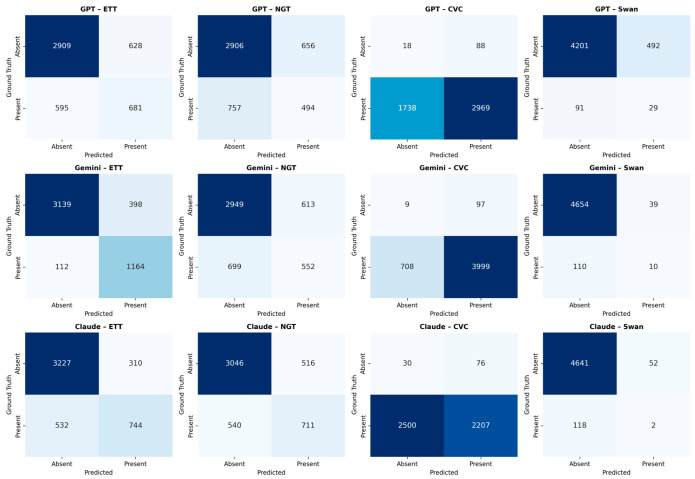
Confusion matrices for device presence detection across models and device types. Rows represent ground truth labels (absent/present); columns represent model predictions. Values indicate case counts.

**Figure 4:**
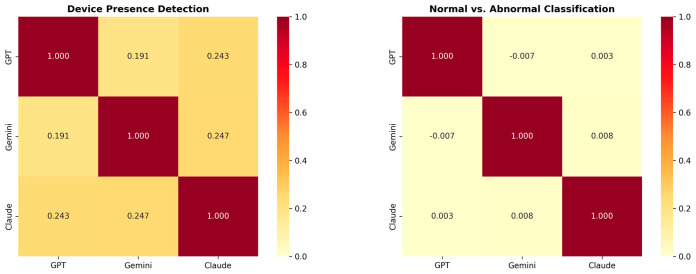
Inter-model agreement heatmap showing mean Cohen’s kappa across device types for device presence detection (**left**) and normal vs. abnormal classification (**right**). Values near zero indicate poor agreement.

**Figure 5:**
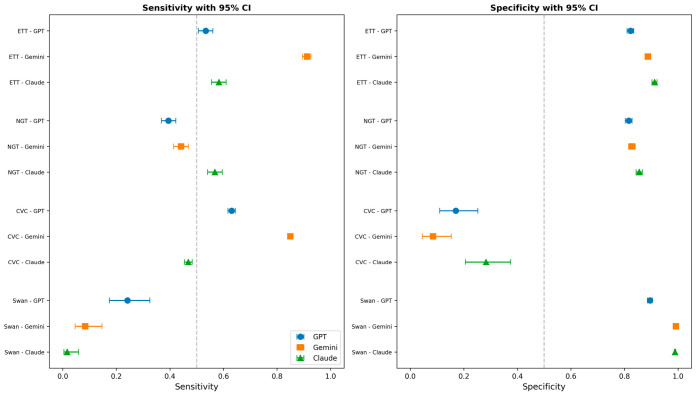
Forest plot of sensitivity (**left**) and specificity (**right**) with 95% confidence intervals for device presence detection across all model–device combinations.

**Figure 6:**
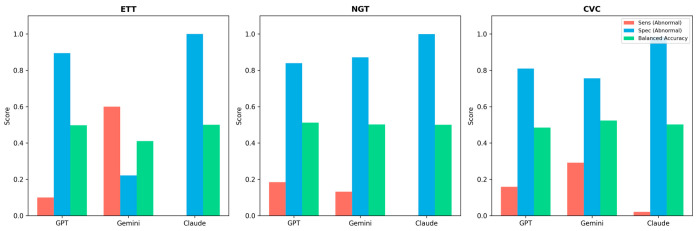
Bar chart comparing normal vs. abnormal classification performance across models for ETT, NGT, and CVC. Red bars = sensitivity for abnormal detection; blue bars = specificity for abnormal; green bars = balanced accuracy.

Among the 4813 analyzed cases, 3 cases (0.06%) had no devices, 3297 cases (68.5%) had a single device, 570 cases (11.8%) had two concurrent devices, and 943 cases (19.6%) had three or more concurrent devices. Subgroup analysis by concurrent device count is provided in Supplementary Table S2. In general, model performance for CVC detection remained stable across subgroups given its high prevalence, while performance for ETT and NGT showed greater variability in multi-device cases (Figure 3, Figure 4, Figure 5 and Figure 6).

### 3.5. Blinded Radiologist Reader Study Results

**Figure 7:**
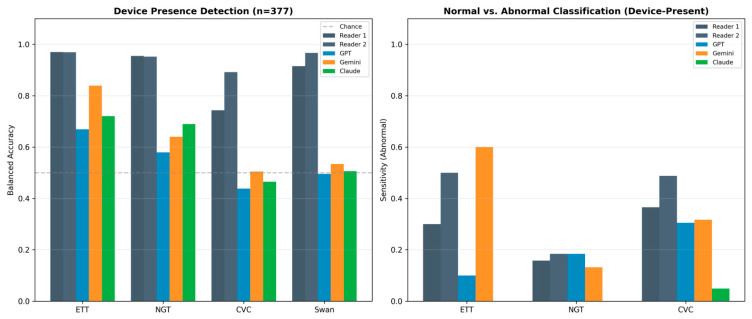
Reader vs. LLM performance on the paired reader study sample (n = 377). (**Left**) Device presence detection balanced accuracy. (**Right**) Sensitivity for abnormal positioning.

**Figure 8:**
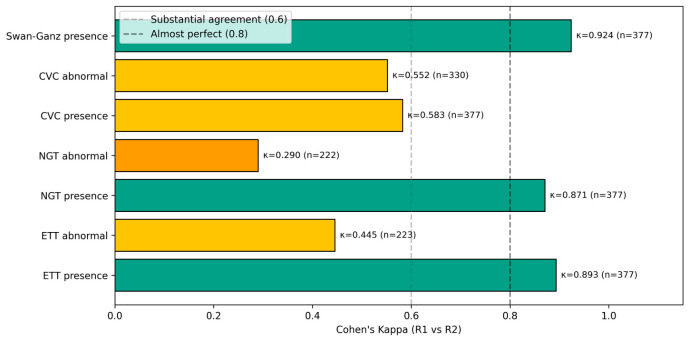
Inter-reader agreement (Cohen’s kappa) between Reader 1 and Reader 2 across device tasks (n = 377; n = device-present cases only for the three classification rows). Bar colour encodes the Landis & Koch interpretation of κ: orange = fair (0.21–0.40), yellow = moderate (0.41–0.60), teal = substantial to almost-perfect (≥0.61). Dashed vertical reference lines mark the substantial-agreement threshold (κ = 0.60) and the almost-perfect threshold (κ = 0.80). Three device-presence agreements (ETT, NGT, Swan–Ganz) reached almost-perfect agreement (κ ≥ 0.80); CVC presence and the ETT and CVC abnormal-position classifications were moderate (κ = 0.45–0.58); NGT abnormal classification reached only fair agreement (κ = 0.29), reflecting the genuine difficulty of malposition adjudication.

Both readers independently completed all 377 cases. Image quality was rated adequate in 257/377 (68%, Reader 1) and 288/377 (76%, Reader 2); 4 cases were rated non-diagnostic by Reader 2 (Table 6 and Figure 7). Inter-reader agreement (Cohen’s kappa) ranged from κ = 0.290 (NGT abnormal) to κ = 0.924 (Swan–Ganz presence), with moderate-to-almost-perfect agreement for all four device-presence tasks (κ = 0.583–0.924). Detailed inter-reader agreement is reported in Supplementary Table S4 and visualized in Figure 8.

Both radiologists numerically outperformed all three LLMs on every device–presence and device–classification task. Examples include ETT presence detection (best LLM: Gemini, balanced accuracy 0.84; both readers 0.97), NGT presence (best LLM: Claude, balanced accuracy 0.69; both readers ≥ 0.95), and Swan–Ganz presence (best LLM: Gemini, balanced accuracy 0.53; both readers ≥ 0.92). For classification, MCC values for the readers were 0.26–0.63 across all device types vs. 0.00–0.10 for the LLMs. Of the 42 paired McNemar comparisons (2 readers × 3 LLMs × 7 device–task pairs), 33 reached statistical significance after Bonferroni correction (*p*_corrected < 0.05) with the reader favored, and 32 reached *p*_corrected < 0.001. The nine non-significant comparisons clustered as expected in cases where the LLM and reader gave similar overall accuracy because the LLM defaulted to the majority class (e.g., Claude classifying nearly all device-present cases as normal, where the gold standard is also predominantly normal due to extreme class imbalance). The reader-vs-LLM comparison is visualized in Figure 7.

### 3.6. External Validation on PadChest Results

Of the 200 PadChest cases successfully processed by all three LLMs, ETT was present in 75 (38%), NGT in 84 (42%), and CVC in 98 (49%). Performance metrics for the three models on PadChest are summarized in Table 7.

**Figure 9:**
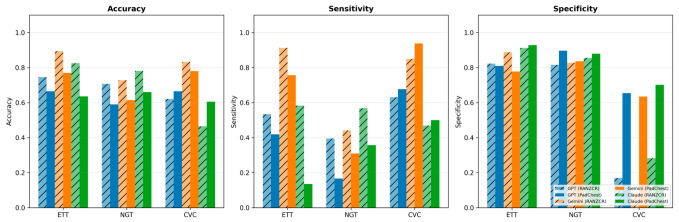
External validation: head-to-head performance on RANZCR (n = 4813) vs. PadChest (n = 200). Accuracy, sensitivity, and specificity for the three LLMs across ETT, NGT, and CVC presence detection. Hatched bars: RANZCR. Solid bars: PadChest.

**Figure 10:**
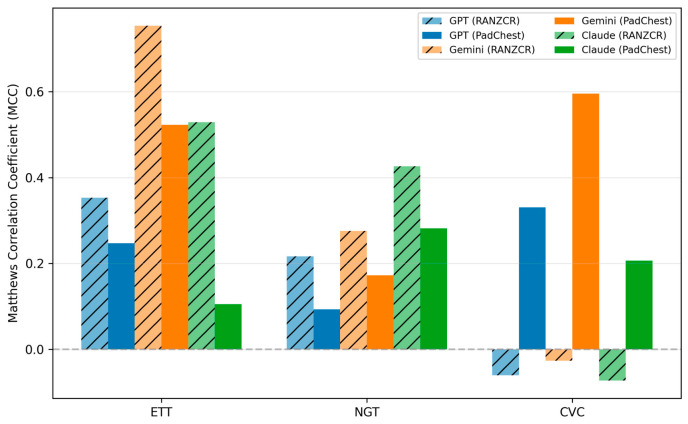
Matthews correlation coefficient (MCC) comparison: RANZCR vs. PadChest. CVC MCC improved markedly on PadChest, confirming the prevalence-driven nature of the original CVC-specificity gap.

A direct head-to-head comparison of RANZCR vs. PadChest is shown in Figure 9 (accuracy, sensitivity, specificity by model and device). Two patterns emerged. First, the qualitative finding—that LLM performance is variable and far below clinical grade for malposition—generalized to PadChest. Second, the previously low CVC specificity in RANZCR (0.085–0.283) rose substantially in PadChest (0.635–0.702), confirming that the original CVC-specificity gap was driven by the extreme CVC prevalence (97.8% in RANZCR) rather than by model failure. Correspondingly, CVC MCC turned positive on PadChest for all three models (0.21–0.60, vs. −0.07 to −0.03 on RANZCR) (Figure 10). Sensitivity for ETT and NGT decreased on PadChest relative to RANZCR (e.g., Claude ETT sensitivity dropped from 0.58 to 0.14), indicating a real domain-shift effect that should be considered before deploying these models in new institutional populations.

### 3.7. Prompt Sensitivity Results

**Figure 11:**
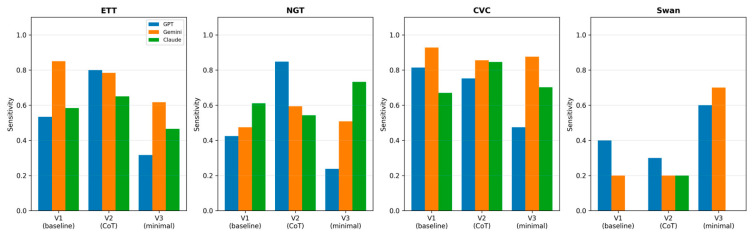
Prompt sensitivity: device-presence sensitivity across three prompt variants (V1 baseline, V2 chain-of-thought, V3 minimal) for the three LLMs (n = 103).

Prompt sensitivity was substantial for all three models, with sensitivity swings of 2–3× across prompt variants for several device–task combinations. GPT-4o demonstrated the most pronounced prompt dependence, with ETT sensitivity ranging from 0.32 (V3 minimal) to 0.80 (V2 chain-of-thought) (2.5× swing) and NGT sensitivity from 0.24 to 0.85 (3.5× swing). Gemini showed somewhat smaller but still meaningful swings (e.g., NGT sensitivity 0.47–0.59); Claude similarly varied (e.g., NGT sensitivity 0.54–0.73). Notably, Swan–Ganz sensitivity for both GPT-4o and Gemini increased markedly with the V3 minimal prompt (0.40 → 0.60 for GPT, 0.20 → 0.70 for Gemini), while V2 chain-of-thought reduced Swan–Ganz sensitivity in some configurations. These findings confirm that single-prompt evaluations of multimodal LLMs may substantially under- or over-estimate performance and that prompt engineering remains an essential consideration in clinical deployment. Per-variant performance is summarized in Figure 11; detailed metrics are provided in Supplementary Table S5.

### 3.8. Repeat-Inference Stability Results

**Figure 12:**
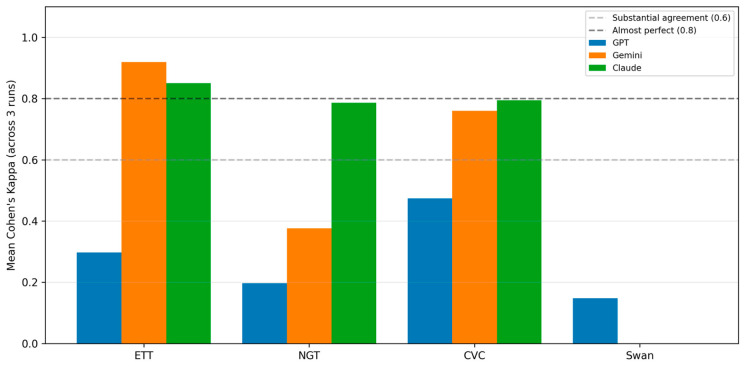
Repeat-inference stability: mean Cohen’s kappa across three independent runs for each model and device (n = 50 cases per run).

Stability across three independent inference runs differed dramatically by model. Mean Cohen’s kappa across the three pairwise run comparisons (Figure 12) showed the following: GPT-4o exhibited fair-to-moderate intra-model stability (mean κ: ETT 0.30, NGT 0.20, CVC 0.47, Swan 0.15), indicating substantial stochasticity even at fixed prompt and inputs. Claude Sonnet 4.6 was the most deterministic (mean κ: ETT 0.85, NGT 0.79, CVC 0.80, Swan 0.00 due to consistent absence). Gemini 3.1 Flash Lite Preview was intermediate (mean κ: ETT 0.92, NGT 0.38, CVC 0.76, Swan 0.00 due to consistent absence). Stability differences likely reflect provider-specific defaults for sampling temperature and other stochastic parameters. The clinical implication is that the same chest radiograph submitted to GPT-4o twice may yield meaningfully different outputs for NGT and ETT—a property that any prospective deployment must accommodate. Detailed run-pair metrics are in Supplementary Table S6.

### 3.9. Error Taxonomy Results

**Figure 13:**
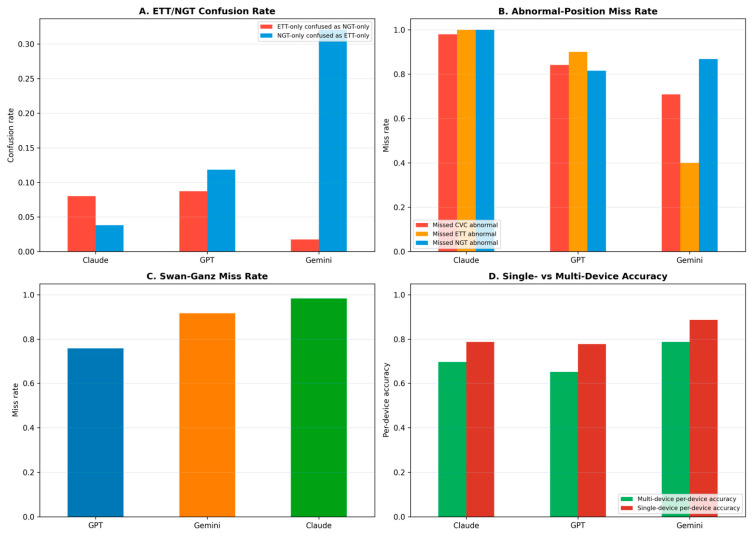
Error taxonomy on the full 4813-case cohort. (**A**) ETT/NGT confusion. (**B**) Abnormal-position miss rate. (**C**) Swan–Ganz miss rate. (**D**) Single- vs. multi-device per-device accuracy.

Systematic error analysis revealed both universal and model-specific failure modes (Figure 13). ETT/NGT confusion was largely model-specific: Gemini misclassified 32% of NGT-only cases as ETT-only, compared with 12% (GPT) and 4% (Claude). Conversely, ETT-only cases were misclassified as NGT-only at low rates across all three models (2–9%). Device hallucination on the three device-absent cases was rare for ETT/NGT/Swan but consistent for CVC (GPT and Gemini hallucinated CVC on all three cases; Claude on one/three), reflecting the prevalence-driven CVC overcalling discussed above. Swan–Ganz catheter detection was a universal weakness: GPT missed 75.8%, Gemini 91.7%, and Claude 98.3% of the 120 Swan-positive cases. Abnormal-position misses were severe and model-specific: Claude missed 100% of ETT-abnormal cases, 100% of NGT-abnormal cases, and 98% of CVC-abnormal cases. GPT and Gemini missed fewer (40–90%) but still alarming proportions. Multi-device degradation was consistent across all three models: per-device accuracy fell by 9–13% on multi-device cases compared with single-device cases (e.g., GPT 0.78 → 0.65, Gemini 0.89 → 0.79, Claude 0.79 → 0.70).

### 3.10. Failure-Case Analysis Results

**Figure 14:**
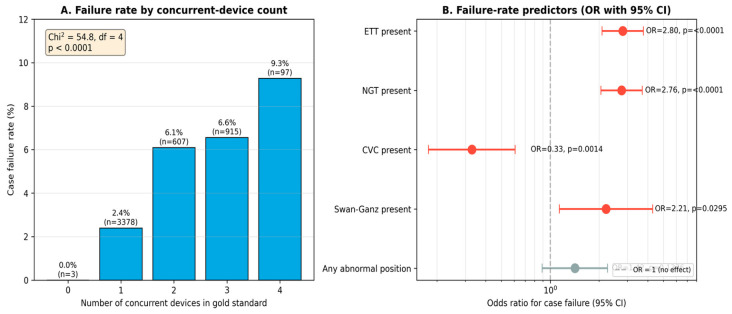
Failure-case analysis. (**A**) The case failure rate increases monotonically with the number of concurrent devices in the gold standard (chi-square test for trend, *p* < 0.0001). (**B**) A forest plot of odds ratios for case failure as a function of presence of each device type and presence of any abnormal positioning. Failures are significantly concentrated in cases with ETT, NGT, or Swan–Ganz devices (red); the CVC association is inverse and reflects extreme prevalence imbalance rather than a protective effect. The presence of any abnormal positioning is not associated with failure (gray, n.s.).

To assess whether the 187 cases excluded due to processing failure (across any of the three models) were a random sample of the cohort or instead concentrated in clinically more complex or abnormal cases—which would bias performance estimates toward easier cases—we examined the distribution of failures with respect to (i) the number of concurrent devices in the gold standard, (ii) the presence of any device with abnormal positioning, and (iii) the presence of each individual device type. Results are visualized in Figure 14.

The case failure rate was strongly and significantly associated with the number of concurrent devices present in the gold standard (Pearson chi-square = 54.8, df = 4, *p* < 0.0001). Failure rate rose monotonically from 0% in device-absent cases (n = 3) to 2.4% with one device (n = 3378), 6.1% with two devices (n = 607), 6.6% with three devices (n = 915), and 9.3% with four concurrent devices (n = 97). Among individual device types, presence of an ETT (odds ratio 2.80; 95% CI 2.09–3.76; *p* < 0.0001), an NGT (OR 2.76; 95% CI 2.06–3.70; *p* < 0.0001), or a Swan–Ganz catheter (OR 2.21; 95% CI 1.14–4.28; *p* = 0.0295) was each significantly associated with a higher case-failure rate. CVC presence was associated with a lower failure rate (OR 0.33; 95% CI 0.18–0.61; *p* = 0.0014), but this finding reflects the extreme prevalence imbalance for CVC (97.6% of cases) rather than a protective effect: the small CVC-absent comparator group (n = 118) had a higher random failure rate.

In contrast, the presence of any abnormal-position device was not significantly associated with failure rate (OR 1.42; 95% CI 0.89–2.26; *p* = 0.138), so the principal finding of poor malposition-detection performance is not undermined by selective failure of abnormal cases. Overall, however, the systematic concentration of failures in cases with more concurrent devices implies that the reported performance estimates may slightly overstate model performance on the clinically more complex multi-device cases that are typical in real ICU practice, and this caveat should be considered when extrapolating to clinical deployment.

## 4. Discussion

In this study, we systematically evaluated and compared the performance of three contemporary multimodal large language models—GPT-4o, Gemini 3.1 Flash Lite Preview, and Claude Sonnet 4.6—in detecting and classifying thoracic devices on chest radiographs using a large, heterogeneous dataset of 4813 cases. Our findings demonstrate that while LLMs achieve variable performance in device presence detection, their ability to identify clinically critical malposition is consistently poor, with balanced accuracy near chance level and MCC values near zero across all models and device types. The principal contribution of this study is therefore not the observation that current general-purpose multimodal LLMs perform poorly on a narrow radiological task—this is consistent with the prior literature—but the structural characterization of *how* and *under which conditions* they fail, including model-specific NGT-as-ETT confusion patterns, prompt-dependent sensitivity swings of 2–3×, intra-model run-to-run instability that differs by an order of magnitude across providers, and a quantified gap relative to expert radiologist reads on the same images.

A key finding is the substantial device-dependent variability in detection performance. ETT detection showed the most balanced performance, with Gemini achieving the highest sensitivity (0.912) and Claude the highest specificity (0.912), corresponding to balanced accuracy values of 0.900 and 0.748, respectively. In contrast, CVC detection demonstrated uniformly high sensitivity but markedly low specificity (range: 0.085–0.283), resulting in negative MCC values for two of three models. This pattern indicates systematic overcalling, likely reflecting both the high prevalence of CVCs in the dataset (97.8% of cases) and the visual similarity between catheters and other linear radiopaque structures. Prior deep learning studies have reported similar trends where models exposed to imbalanced datasets exhibit inflated sensitivity at the expense of specificity [4,5]. The external validation on PadChest provided direct evidence in support of the prevalence interpretation: at PadChest’s much lower CVC prevalence (49%), CVC specificity rose to 0.63–0.70, and CVC MCC turned positive (0.21–0.60) for all three models. This confirms that the original CVC-specificity finding was driven by base-rate effects rather than by an inherent model failure on CVC recognition.

The detection of Swan–Ganz catheters was characterized by very low sensitivity (range: 0.017–0.242) despite high specificity, highlighting the difficulty of identifying rare devices. This pattern, confirmed by MCC values near zero (0.008–0.117), suggests that multimodal LLMs are sensitive to prevalence and visual familiarity. A recent evaluation of multimodal LLMs in pneumothorax detection similarly demonstrated reduced sensitivity for less frequent findings [26], and additional studies have emphasized that performance degradation is more pronounced for rare clinical entities [10,27].

The most clinically significant finding is the consistently poor performance in distinguishing normal from abnormal positioning. MCC values ranged from −0.038 to 0.028, and balanced accuracy ranged from 0.411 to 0.527, indicating that no model achieved discrimination meaningfully above chance. Claude, in particular, showed near-zero sensitivity for abnormal positioning across all device types, suggesting an extreme bias toward normal classification. This “normality bias” likely reflects uncertainty-avoidance behavior inherent in the models’ training, where the default or “safe” response is to classify devices as normally positioned. A recent study on generative AI for chest radiograph interpretation in emergency department settings similarly observed that LLMs tend toward conservative interpretations with reduced sensitivity for acute findings [28]. Beyond chest radiography, sub-clinical LLM performance has been documented in adjacent imaging-based specialties as well—for example, GPT-3.5 evaluated on the Polish national specialty examination in nuclear medicine achieved only 56% accuracy, below the 60% pass threshold [29], reinforcing that current general-purpose LLMs often do not meet the standards expected for autonomous use in specialty medical contexts.

In direct paired comparison, the present reader study (Section 3.5) demonstrated that two board-certified radiologists outperformed all three LLMs by a wide margin on the same images: reader MCC values for normal-vs-abnormal classification ranged from 0.26 to 0.63, an order of magnitude above the 0.00–0.10 range observed for the LLMs. This reframes the negative finding from “LLMs perform poorly at malposition detection” to “LLMs perform poorly relative to the human standard on the same task on the same images,” strengthening the safety argument for non-deployment in autonomous workflows. The contrast also aligns with published benchmarks of task-specific deep learning systems for ETT misposition that achieve up to 89% accuracy in AI-assisted reader workflows [16], reinforcing the gap between specialist and generalist AI for this task.

A further novel contribution of this study is the systematic assessment of inter-model agreement, which revealed striking findings. Fleiss’ kappa for device presence ranged from 0.005 to 0.383 (poor to fair), while for classification, it was uniformly negative (−0.280 to −0.025), indicating agreement worse than chance. This suggests that the three models employ fundamentally different internal reasoning strategies, arriving at different conclusions for the same cases. The lack of convergence is particularly concerning for clinical applications, as it implies that the choice of model may substantially influence diagnostic output. Comparable inter-model variability has been observed in comparative studies of LLMs on diagnostic radiology cases, where no single model consistently outperformed others [13,14]. The prompt-sensitivity and stability analyses (Section 3.7 and Section 3.8) further deepen these observations. Sensitivity swings of 2–3× were observed across three prompt variants for the same model and same images, and intra-model run-to-run agreement varied from κ = 0.20 (GPT-4o on NGT) to κ = 0.85 (Claude on ETT) using each provider’s default sampling parameters. These two findings imply that any individual evaluation point in the present main analysis represents a single draw from a distribution of possible outputs that depends on prompt phrasing and stochastic sampling. They also imply that any clinical deployment must contend with non-deterministic behavior—particularly for GPT-4o—and that prompt engineering effort can move performance metrics by clinically meaningful amounts. The error taxonomy (Section 3.9) additionally identified specific failure patterns: model-specific NGT-as-ETT confusion in Gemini (32%), universal Swan–Ganz blindness, and severe abnormal-position misses (notably 100% in Claude for ETT and NGT). Together, these characterizations transform the headline negative result from “current LLMs perform poorly at this task” into a structured safety benchmark identifying the specific conditions and failure modes under which they fail.

The limitations of spatial reasoning in multimodal LLMs explain much of the observed performance deficit. Unlike conventional convolutional neural networks optimized for localization, LLMs rely on vision–language alignment and implicit reasoning rather than precise geometric analysis. Tasks such as assessing ETT tip position relative to the carina, confirming NGT passage into the stomach, or determining optimal CVC tip location at the cavoatrial junction require fine-grained spatial awareness that current multimodal architectures do not reliably provide [12,30]. Device-specific complexity further explains the observed differences: ETT evaluation depends on relatively well-defined landmarks, whereas NGT assessment requires continuous trajectory tracking, and CVC evaluation is complicated by multiple insertion routes and acceptable tip positions [14]. Recent reviews on AI applications for thoracic imaging have emphasized that while foundation models show broad capabilities, task-specific spatial reasoning remains a critical bottleneck [18].

An important methodological consideration is the role of prompt design. All models were evaluated using a single standardized prompt, and prior work has shown that LLM outputs are sensitive to prompt formulation and contextual framing [9,31]. Alternative prompting strategies—such as chain-of-thought reasoning, few-shot examples with annotated images, or structured multi-step evaluation protocols—may improve performance and should be explored in future studies. Additionally, the cost and latency of API-based inference represent practical barriers to clinical deployment that warrant consideration. The evolving landscape of multimodal LLMs in radiology, with rapid model iterations and expanding capabilities, suggests that periodic re-evaluation will be necessary as newer model versions become available [13].

This study has several limitations. First, the extreme class imbalance in abnormal positioning—particularly for ETT (10 abnormal out of 1276 present, 0.8%) and NGT (38 out of 1251, 3.0%)—limits the statistical power and reliability of sensitivity estimates for abnormal detection. The wide confidence intervals for ETT abnormal sensitivity (e.g., 0.000–0.278 for Claude, 0.313–0.832 for Gemini) reflect this constraint, and the results for ETT classification should be interpreted with particular caution. We re-emphasize that the absolute number of abnormal ETT (n = 10) and abnormal NGT (n = 38) cases in the RANZCR cohort is small in absolute terms; the wide 95% confidence intervals on malposition sensitivity (e.g., 0.000–0.278 to 0.313–0.832 for ETT abnormal across models) reflect this and substantially limit the precision of any malposition-detection conclusion drawn from these device categories. Conclusions about ETT and NGT malposition are therefore best read as descriptive findings on a small abnormal-case set, not as precision estimates of true population sensitivity. Second, the binary classification scheme simplifies a complex clinical spectrum and may not capture borderline or context-dependent cases. Third, all models were accessed via commercial APIs with default settings; model versions and underlying architectures may change over time, potentially affecting reproducibility. Newer versions across all three providers have been released since the analyses were conducted (e.g., later GPT-4 family snapshots, subsequent Gemini Flash/Pro releases beyond 3.1 Flash Lite Preview, and Claude Opus 4.x), and the specific versions used here may not represent the current state of the art. The exact model IDs are documented in Section 2.4 to enable comparison with future evaluations. Fourth, the dataset is large and heterogeneous, and the PadChest external validation supports generalizability of the device-presence findings to an independent population (granular position labels were not available in PadChest, so external validation of malposition detection was not possible and remains an open question for future work). Fifth, real-world clinical deployment of commercial LLM APIs would require frameworks compliant with HIPAA, GDPR, and institution-specific data-protection regulations. The three models also represent distinct architectural tiers and release timelines, so direct apples-to-apples comparison is an approximation. The present study used only publicly available, fully de-identified imaging datasets and does not establish the regulatory compatibility of any of the evaluated providers for clinical deployment.

From a clinical perspective, these findings suggest that current multimodal LLMs are not yet suitable for autonomous evaluation of thoracic devices. However, their relatively high specificity for certain devices indicates potential utility as adjunct screening tools—for example, confirming clearly normal placements or supporting triage workflows. Finally, we emphasize that the three systems evaluated are general-purpose multimodal LLMs designed for broad use rather than diagnostic AI systems optimized for radiology or carrying regulatory clearance (e.g., FDA 510(k) or CE-mark). The poor malposition-detection performance we report does not generalize to or imply anything about purpose-built, regulator-approved thoracic-device AI systems, which are evaluated separately in dedicated reader studies (e.g., [7,16]). Our findings characterize the safety profile of general-purpose multimodal LLMs in this specific clinical task and do not constitute a comparison against the current state of the art in specialist medical AI [5,16]. Future research should focus on improving spatial reasoning capabilities, potentially through hybrid architectures combining LLMs with dedicated computer vision systems, incorporating anatomical constraints and domain-specific fine-tuning. Prospective validation studies will be essential to determine the clinical impact of these systems.

## 5. Conclusions

In this large-scale comparative study, multimodal large language models demonstrated variable performance in thoracic device detection on chest radiographs, with device presence detection ranging from acceptable for ETT (balanced accuracy up to 0.900) to poor for Swan–Ganz catheters (balanced accuracy ~0.50). Critically, all models failed to reliably identify abnormal device positioning, with MCC values near zero and balanced accuracy at chance level across all device types. Inter-model agreement was poor to slight for device detection and worse than chance for classification. In paired comparison, two board-certified radiologists numerically outperformed all three LLMs on every device–task pair, with the superiority reaching statistical significance after Bonferroni correction in 33 of 42 paired comparisons. External validation on PadChest (presence detection only; PadChest does not provide granular position labels) demonstrated meaningful domain-shift effects on presence detection. Furthermore, prompt-sensitivity and intra-model stability analyses showed that LLM outputs are substantially modulated by prompt phrasing and stochastic sampling, while the error taxonomy identified model-specific failure modes (e.g., Gemini’s NGT-as-ETT confusion, Claude’s 100% miss of ETT/NGT abnormal positioning). These findings indicate that current multimodal LLMs may serve a limited supportive role in device presence screening but are not yet reliable for the clinically critical task of malposition detection.

## Figures and Tables

**Table 3:** Normal vs. abnormal classification performance with 95% confidence intervals (device-present cases).

Model	Device	N (Abn)	Accuracy (95% CI)	Sens Abn (95% CI)	Spec Abn (95% CI)	Bal. Acc.	MCC
GPT	ETT	1276 (10)	0.889 (0.870–0.905)	0.100 (0.018–0.404)	0.895 (0.877–0.911)	0.497	−0.001
GPT	NGT	1251 (38)	0.819 (0.797–0.840)	0.184 (0.092–0.334)	0.839 (0.818–0.859)	0.512	0.011
GPT	CVC	4707 (347)	0.762 (0.749–0.774)	0.159 (0.124–0.201)	0.810 (0.798–0.821)	0.484	−0.021
Gemini	ETT	1276 (10)	0.224 (0.202–0.248)	0.600 (0.313–0.832)	0.221 (0.199–0.245)	0.411	−0.038
Gemini	NGT	1251 (38)	0.849 (0.828–0.868)	0.132 (0.058–0.273)	0.871 (0.851–0.889)	0.501	0.002
Gemini	CVC	4707 (347)	0.722 (0.709–0.734)	0.291 (0.246–0.341)	0.756 (0.743–0.768)	0.524	0.028
Claude	ETT	1276 (10)	0.992 (0.986–0.996)	0.000 (0.000–0.278)	1.000 (0.997–1.000)	0.500	0.000
Claude	NGT	1251 (38)	0.969 (0.958–0.977)	0.000 (0.000–0.092)	0.999 (0.995–1.000)	0.500	−0.005
Claude	CVC	4707 (347)	0.913 (0.904–0.920)	0.020 (0.010–0.041)	0.984 (0.980–0.987)	0.502	0.008

N (Abn) = total device-present cases (abnormal cases); Bal. Acc. = balanced accuracy; MCC = Matthews correlation coefficient.

**Table 4:** Cochran’s Q test and pairwise McNemar tests with Bonferroni correction for normal vs. abnormal classification.

Device	Cochran’s Q	Cochran *p*	Comparison	*p* (Raw)	*p* (Corrected)	Better
ETT	1668.6	<0.001	GPT vs. Gemini	<0.001	<0.001	GPT
ETT	1668.6	<0.001	GPT vs. Claude	<0.001	<0.001	Claude
ETT	1668.6	<0.001	Gemini vs. Claude	<0.001	<0.001	Claude
NGT	171.0	<0.001	GPT vs. Gemini	0.046	0.411	Gemini
NGT	171.0	<0.001	GPT vs. Claude	<0.001	<0.001	Claude
NGT	171.0	<0.001	Gemini vs. Claude	<0.001	<0.001	Claude
CVC	728.4	<0.001	GPT vs. Gemini	<0.001	<0.001	GPT
CVC	728.4	<0.001	GPT vs. Claude	<0.001	<0.001	Claude
CVC	728.4	<0.001	Gemini vs. Claude	<0.001	<0.001	Claude

**Table 5:** Inter-model agreement: Cohen’s kappa (pairwise) and Fleiss’ kappa (three-model).

Task	Device	Type	Comparison	Kappa
Presence	ETT	Cohen	GPT vs. Gemini	0.357
Presence	ETT	Cohen	GPT vs. Claude	0.294
Presence	ETT	Cohen	Gemini vs. Claude	0.501
Presence	ETT	Fleiss	All models	0.383
Presence	NGT	Cohen	GPT vs. Gemini	0.155
Presence	NGT	Cohen	GPT vs. Claude	0.258
Presence	NGT	Cohen	Gemini vs. Claude	0.238
Presence	NGT	Fleiss	All models	0.217
Presence	CVC	Cohen	GPT vs. Gemini	0.233
Presence	CVC	Cohen	GPT vs. Claude	0.385
Presence	CVC	Cohen	Gemini vs. Claude	0.181
Presence	CVC	Fleiss	All models	0.230
Presence	Swan	Cohen	GPT vs. Gemini	0.017
Presence	Swan	Cohen	GPT vs. Claude	0.036
Presence	Swan	Cohen	Gemini vs. Claude	0.068
Presence	Swan	Fleiss	All models	0.005
Classification	ETT	Cohen	GPT vs. Gemini	0.011
Classification	ETT	Cohen	GPT vs. Claude	0.000
Classification	ETT	Cohen	Gemini vs. Claude	0.000
Classification	ETT	Fleiss	All models	−0.280
Classification	NGT	Cohen	GPT vs. Gemini	−0.045
Classification	NGT	Cohen	GPT vs. Claude	−0.002
Classification	NGT	Cohen	Gemini vs. Claude	0.011
Classification	NGT	Fleiss	All models	−0.047
Classification	CVC	Cohen	GPT vs. Gemini	0.012
Classification	CVC	Cohen	GPT vs. Claude	0.009
Classification	CVC	Cohen	Gemini vs. Claude	0.014
Classification	CVC	Fleiss	All models	−0.025

Interpretation (Landis and Koch): <0 poor, 0–0.20 slight, 0.21–0.40 fair, 0.41–0.60 moderate, 0.61–0.80 substantial, 0.81–1.00 almost perfect.

**Table 6:** Reader vs. three LLMs’ performance on the reader-study subset (n = 377).

Rater	Device	Presence Bal Acc	Presence MCC	Classif Bal Acc	Classif MCC
Reader 1	ETT	0.970	0.927	0.641	0.336
Reader 1	NGT	0.955	0.897	0.574	0.302
Reader 1	CVC	0.743	0.382	0.620	0.263
Reader 2	ETT	0.970	0.923	0.748	0.635
Reader 2	NGT	0.952	0.884	0.590	0.365
Reader 2	CVC	0.892	0.482	0.706	0.462
GPT	ETT	0.669	0.327	0.483	−0.019
GPT	NGT	0.579	0.158	0.503	0.005
GPT	CVC	0.438	−0.062	0.535	0.067
Gemini	ETT	0.839	0.685	0.431	−0.062
Gemini	NGT	0.640	0.277	0.498	−0.004
Gemini	CVC	0.504	0.007	0.551	0.101
Claude	ETT	0.720	0.424	0.500	0.000
Claude	NGT	0.690	0.362	0.500	0.000
Claude	CVC	0.465	−0.029	0.512	0.058
Reader 1	Swan	0.915	0.888	N/A	N/A
Reader 2	Swan	0.967	0.960	N/A	N/A
GPT	Swan	0.495	−0.008	N/A	N/A
Gemini	Swan	0.534	0.154	N/A	N/A
Claude	Swan	0.506	0.027	N/A	N/A

Bal Acc = balanced accuracy; MCC = Matthews correlation coefficient; N/A = Swan–Ganz classification not assessed.

**Table 7:** External validation on PadChest (n = 200): device presence detection.

Model	Device	Accuracy (95% CI)	Sensitivity (95% CI)	Specificity (95% CI)	Bal Acc	MCC
GPT	ETT	0.665 (0.597–0.727)	0.419 (0.313–0.533)	0.810 (0.732–0.869)	0.614	0.247
GPT	NGT	0.590 (0.521–0.656)	0.167 (0.102–0.261)	0.897 (0.828–0.940)	0.532	0.093
GPT	CVC	0.665 (0.597–0.727)	0.677 (0.578–0.762)	0.654 (0.558–0.738)	0.665	0.331
Gemini	ETT	0.770 (0.707–0.823)	0.757 (0.648–0.840)	0.778 (0.698–0.842)	0.767	0.523
Gemini	NGT	0.615 (0.546–0.680)	0.310 (0.221–0.415)	0.836 (0.758–0.893)	0.573	0.172
Gemini	CVC	0.780 (0.718–0.832)	0.938 (0.870–0.971)	0.635 (0.539–0.721)	0.786	0.595
Claude	ETT	0.635 (0.566–0.699)	0.135 (0.075–0.231)	0.929 (0.870–0.962)	0.532	0.105
Claude	NGT	0.660 (0.592–0.722)	0.357 (0.263–0.464)	0.879 (0.808–0.927)	0.618	0.282
Claude	CVC	0.605 (0.536–0.670)	0.500 (0.402–0.598)	0.702 (0.608–0.781)	0.601	0.206

## Data Availability

The data were obtained from the third-party RANZCR CLiP dataset (https://www.kaggle.com/c/ranzcr-clip-catheter-line-classification; accessed on 22 March 2026) and are publicly available from the Kaggle competition page. The PadChest dataset is publicly available from the BIMCV BSC Nextcloud repository (https://bimcv.cipf.es/bimcv-projects/padchest/; accessed on 22 March 2026). The derived data generated in this study are available from the corresponding author upon reasonable request.

## Supplementary Materials

The following supporting information can be downloaded at https://www.mdpi.com/article/10.3390/diagnostics16111602/s1, Confusion matrices for all model–device combinations (Table S1); Subgroup analysis by concurrent device count (Table S2); STARD-AI checklist (Table S3, extends STARD 2015 with 14 AI-extension items). Reader-study additional metrics including inter-reader agreement and paired McNemar comparisons (Table S4); Per-variant prompt-sensitivity metrics (Table S5); Intra-model stability run-pair metrics (Table S6); Per-stratum error-taxonomy counts (Table S7); The three prompt variants V1/V2/V3 in full (Supplementary Methods S1).
