# Supplementary material for: Performance of Multimodal Large Language Models in Detection and Position Assessment of Thoracic Devices on Chest Radiographs
Source: Diagnostics (Basel). 2026 May 23;16(11):1602. doi: 10.3390/diagnostics16111602 (PMC13257059; doi:10.3390/diagnostics16111602)
Supplement: Supplementary file 1 [file diagnostics-16-01602-s001.zip › Methods_S1_Prompt_Variants.pdf]

Supplementary Methods S1

Full Text of the Three Prompt Variants Used in the Prompt Sensitivity Analysis

Three prompt variants were tested on a stratified subset of 103 RANZCR cases (Section 2.9). V1 is the baseline prompt used in the main analysis. V2 explicitly instructs chain-of-thought reasoning. V3 is a minimal/concise variant. All three were submitted with the same model parameters (max_tokens=500, default temperature) to all three models.

V1: Baseline Prompt

Analyze the provided chest X-ray and classify the presence and position of the following devices.

DEFINITIONS:
- ETT: Endotracheal Tube.
- NGT: Nasogastric Tube.
- CVC: Central Venous Catheter.
- Swan–Ganz Catheter: Pulmonary artery catheter.

CLASSIFICATION RULES:
For each device:
- "Normal" = correctly positioned or acceptable.
- "Abnormal" = clearly malpositioned or unsafe.

GENERAL RULES:
- If a device is NOT present → all its categories must be 0.
- If a device is visible, you MUST classify it and assign exactly one category as 1.
- Search carefully for each device; do not miss a device.
- For each device (ETT, NGT, CVC), ONLY ONE of [Abnormal, Normal] can be 1.
- Devices are independent; multiple devices can be present and have 1 simultaneously.
- Swan–Ganz is binary: 1 if present, 0 if absent.

OUTPUT FORMAT:
Return ONLY valid JSON.
Do not include any text before or after the JSON.

```
{
  "ETT - Abnormal": 0,
  "ETT - Normal": 0,
  "NGT - Abnormal": 0,
  "NGT - Normal": 0,
  "CVC - Abnormal": 0,
  "CVC - Normal": 0,
  "Swan Ganz Catheter Present": 0
}
```

V2: Chain-of-Thought Prompt

Analyze the provided chest X-ray and classify the presence and position of the following devices.

DEFINITIONS:
- ETT: Endotracheal Tube (correct: tip 3-7 cm above carina).
- NGT: Nasogastric Tube (correct: passing through esophagus into stomach).
- CVC: Central Venous Catheter (correct: tip near cavoatrial junction).
- Swan–Ganz Catheter: Pulmonary artery catheter.

REASONING APPROACH:
Before producing the JSON, reason step by step about each device:
1. ETT: Is a radiopaque tube visible in the trachea? If yes, where is its tip relative to the carina?
2. NGT: Is a tube passing through the esophagus? Does it follow the expected path into the stomach, or is it coiled, in a bronchus, or terminating in the esophagus?
3. CVC: Are any central venous catheters visible? Identify the insertion route (subclavian, jugular, PICC, umbilical). Where is the tip relative to the cavoatrial junction?
4. Swan–Ganz: Is a catheter visible crossing the right heart into the pulmonary artery?

After your reasoning, produce ONLY valid JSON in the format below. Do not include the reasoning in the output.

CLASSIFICATION RULES:
- "Normal" = correctly positioned or clinically acceptable.
- "Abnormal" = clearly malpositioned or unsafe.
- For each device (ETT, NGT, CVC), exactly ONE of [Abnormal, Normal] can be 1 IF the device is present; otherwise both 0.
- Swan–Ganz is binary (present = 1, absent = 0).

OUTPUT FORMAT (JSON only, no prose):

```
{
  "ETT - Abnormal": 0,
  "ETT - Normal": 0,
  "NGT - Abnormal": 0,
  "NGT - Normal": 0,
  "CVC - Abnormal": 0,
  "CVC - Normal": 0,
  "Swan Ganz Catheter Present": 0
}
```

V3: Minimal/Concise Prompt

Identify thoracic devices on this chest X-ray. For each device, set Abnormal=1 if visibly malpositioned, Normal=1 if visibly correctly positioned, or both 0 if absent. Swan–Ganz: 1 if present.

Reply with JSON only:

```
{"ETT - Abnormal": 0, "ETT - Normal": 0, "NGT - Abnormal": 0, "NGT - Normal": 0, "CVC - Abnormal": 0, "CVC - Normal": 0, "Swan Ganz Catheter Present": 0}
```
