# Supplementary material for: Performance of Multimodal Large Language Models in Detection and Position Assessment of Thoracic Devices on Chest Radiographs
Source: Diagnostics (Basel). 2026 May 23;16(11):1602. doi: 10.3390/diagnostics16111602 (PMC13257059; doi:10.3390/diagnostics16111602)
Supplement: Supplementary file 1 [file diagnostics-16-01602-s001.zip › Table_S1_Confusion_Matrices.pdf]

Supplementary Table S1

Confusion Matrices for Device Presence Detection and Normal vs. Abnormal Classification

Table S1a. Confusion matrices for device presence detection (n=4813).

Values represent case counts. Columns are model predictions; rows are gold-standard labels.

Model	Device	TN	FP	FN	TP	Total
GPT	ETT	2909	628	595	681	4813
GPT	NGT	2906	656	757	494	4813
GPT	CVC	18	88	1738	2969	4813
GPT	Swan	4201	492	91	29	4813
Gemini	ETT	3139	398	112	1164	4813
Gemini	NGT	2949	613	699	552	4813
Gemini	CVC	9	97	708	3999	4813
Gemini	Swan	4654	39	110	10	4813
Claude	ETT	3227	310	532	744	4813
Claude	NGT	3046	516	540	711	4813
Claude	CVC	30	76	2500	2207	4813
Claude	Swan	4641	52	118	2	4813

TN = true negative; FP = false positive; FN = false negative; TP = true positive.

Table S1b. Confusion matrices for normal vs. abnormal classification (device-present cases only).

Positive class = abnormal. Only cases where the device was truly present in the gold standard are included.

Model	Device	TN	FP	FN	TP	Total
GPT	ETT	1133	133	9	1	1276
GPT	NGT	1018	195	31	7	1251
GPT	CVC	3530	830	292	55	4707
Gemini	ETT	280	986	4	6	1276
Gemini	NGT	1057	156	33	5	1251
Gemini	CVC	3296	1064	246	101	4707
Claude	ETT	1266	0	10	0	1276
Claude	NGT	1212	1	38	0	1251
Claude	CVC	4289	71	340	7	4707

TN = correctly classified as normal; FP = normal classified as abnormal; FN = abnormal classified as normal; TP = correctly classified as abnormal.
