# Supplementary material for: Performance of Multimodal Large Language Models in Detection and Position Assessment of Thoracic Devices on Chest Radiographs
Source: Diagnostics (Basel). 2026 May 23;16(11):1602. doi: 10.3390/diagnostics16111602 (PMC13257059; doi:10.3390/diagnostics16111602)
Supplement: Supplementary file 1 [file diagnostics-16-01602-s001.zip › Table_S2_Subgroup_Analysis.pdf]

Supplementary Table S2

Subgroup Analysis: Device Presence Detection by Number of Concurrent Devices

Table S2. Device presence detection performance stratified by number of concurrent devices.

Cases were stratified by the total number of devices present in the gold standard (0, 1, 2, or 3+ devices).

Model	Group	N cases	Device	Accuracy	Sensitivity	Specificity	F1
GPT	0	3	ETT	0.667	N/A	0.667	0.000
GPT	0	3	NGT	0.333	N/A	0.333	0.000
GPT	0	3	CVC	0.000	N/A	0.000	0.000
GPT	0	3	Swan	0.667	N/A	0.667	0.000
GPT	1	3297	ETT	0.829	0.348	0.833	0.028
GPT	1	3297	NGT	0.834	0.375	0.837	0.032
GPT	1	3297	CVC	0.520	0.523	0.277	0.682
GPT	1	3297	Swan	0.929	N/A	0.929	0.000
GPT	2	570	ETT	0.640	0.591	0.700	0.643
GPT	2	570	NGT	0.454	0.347	0.566	0.394
GPT	2	570	CVC	0.758	0.831	0.089	0.861
GPT	2	570	Swan	0.812	0.273	0.834	0.101
GPT	3+	943	ETT	0.519	0.519	0.333	0.683
GPT	3+	943	NGT	0.415	0.410	1.000	0.582
GPT	3+	943	CVC	0.892	0.892	N/A	0.943
GPT	3+	943	Swan	0.743	0.235	0.802	0.160
Gemini	0	3	ETT	0.667	N/A	0.667	0.000
Gemini	0	3	NGT	1.000	N/A	1.000	0.000
Gemini	0	3	CVC	0.000	N/A	0.000	0.000
Gemini	0	3	Swan	1.000	N/A	1.000	0.000
Gemini	1	3297	ETT	0.925	0.783	0.926	0.127
Gemini	1	3297	NGT	0.837	0.458	0.840	0.039
Gemini	1	3297	CVC	0.790	0.799	0.128	0.882
Gemini	1	3297	Swan	0.997	N/A	0.997	0.000
Gemini	2	570	ETT	0.674	0.895	0.405	0.751
Gemini	2	570	NGT	0.565	0.447	0.688	0.512
Gemini	2	570	CVC	0.849	0.936	0.054	0.918
Gemini	2	570	Swan	0.951	0.045	0.987	0.067
Gemini	3+	943	ETT	0.919	0.921	0.333	0.958
Gemini	3+	943	NGT	0.441	0.439	0.714	0.609
Gemini	3+	943	CVC	0.977	0.977	N/A	0.988
Gemini	3+	943	Swan	0.881	0.092	0.973	0.138
Claude	0	3	ETT	1.000	N/A	1.000	0.000
Claude	0	3	NGT	1.000	N/A	1.000	0.000
Claude	0	3	CVC	0.667	N/A	0.667	0.000
Claude	0	3	Swan	1.000	N/A	1.000	0.000
Claude	1	3297	ETT	0.927	0.478	0.930	0.084
Claude	1	3297	NGT	0.888	0.375	0.892	0.047
Claude	1	3297	CVC	0.344	0.344	0.340	0.508
Claude	1	3297	Swan	0.993	N/A	0.993	0.000
Claude	2	570	ETT	0.640	0.597	0.693	0.646
Claude	2	570	NGT	0.440	0.440	0.441	0.445
Claude	2	570	CVC	0.646	0.693	0.214	0.779
Claude	2	570	Swan	0.944	0.000	0.982	0.000
Claude	3+	943	ETT	0.580	0.581	0.333	0.734

Claude	3+	943	NGT	0.610	0.613	0.143	0.757
Claude	3+	943	CVC	0.778	0.778	N/A	0.875
Claude	3+	943	Swan	0.879	0.020	0.979	0.034

Group = number of concurrent devices in gold standard; N/A = metric not calculable (no positive or no negative cases in subgroup).
