# Supplementary material for: Performance of Multimodal Large Language Models in Detection and Position Assessment of Thoracic Devices on Chest Radiographs
Source: Diagnostics (Basel). 2026 May 23;16(11):1602. doi: 10.3390/diagnostics16111602 (PMC13257059; doi:10.3390/diagnostics16111602)
Supplement: Supplementary file 1 [file diagnostics-16-01602-s001.zip › Table_S3_STARD_AI_Checklist.pdf]

Supplementary Table S3

STARD-AI Checklist: Standards for Reporting Diagnostic Accuracy Studies — AI extension

This study followed the STARD 2015 reporting framework (Bossuyt et al. 2015), extended with the STARD-AI reporting guideline for diagnostic accuracy studies using artificial intelligence (Sounderajah et al., Nat. Med. 2025;31:3283–3289). The published STARD-AI checklist adds 18 new or modified items to the STARD 2015 baseline, covering dataset and training-data provenance, AI index-test specification (model architecture, version, parameters), evaluation methodology and validation frameworks, algorithmic bias and fairness across subgroups, generalizability and applicability, explainability and interpretability, uncertainty and calibration, human–AI workflow integration, and regulatory/ethical considerations. Because STARD-AI was designed primarily for classical (task-specific) diagnostic AI rather than generative multimodal LLMs, our mapping below also includes items that are specifically relevant to LLM evaluation (e.g., prompt design, prompt sensitivity, intra-model stability) and that we believe a reader of an LLM-based diagnostic-accuracy study would expect to see documented.

Table S3. STARD-AI Checklist.

Section and Topic	No.	Item	Reported on page / location
**TITLE/ABSTRACT**	1	Identification as a study of diagnostic accuracy using at least one measure of accuracy	Title; Abstract
**ABSTRACT**	2	Structured summary of study design, methods, results, and conclusions	Abstract (Background, Methods, Results, Conclusions)
**INTRODUCTION**	3	Scientific and clinical background, including the intended use and clinical role of the index test	Section 1, paragraphs 1–6
**INTRODUCTION**	4	Study objectives and hypotheses	Section 1, final paragraph; Section 2.6 (pre-specified H0)
**METHODS – Study design**	5	Prospective vs. retrospective data collection	Section 2.1 (retrospective; public dataset)
**METHODS – Participants**	6	Eligibility criteria	Section 2.1
**METHODS – Participants**	7	On what basis participants were identified	Section 2.1
**METHODS – Participants**	8	Where and when participants were identified	Section 2.1 (RANZCR CLiP); Section 2.4 (March 15–31, 2026 access window)
**METHODS – Participants**	9	Whether participants formed a consecutive, random, or convenience series	Section 2.1 (simple random sample without replacement, fixed seed)
**METHODS – Test methods**	10a	Index test, in sufficient detail to allow replication	Sections 2.4–2.5; Supplementary Methods S1; Supplementary Code S1
**METHODS – Test methods**	10b	Reference standard, in sufficient detail to allow replication	Section 2.1 (RANZCR CLiP expert annotations); Section 2.8 (PadChest device-presence labels)
**METHODS – Test methods**	11	Rationale for choosing the reference standard	Section 2.1 (public expert-labeled dataset); Section 2.8 (PadChest rationale,

			presence-only scope)
**METHODS – Test methods**	12a	Definition and rationale for test positivity cut-offs / result categories of the index test	Section 2.3 (binary classification, pre-specified); Section 2.5 (prompt rules)
**METHODS – Test methods**	12b	Definition and rationale for cut-offs / categories of the reference standard	Section 2.3 (binary normal/abnormal per expert annotation, pre-specified)
**METHODS – Test methods**	13a	Whether clinical information and reference standard results were available to readers of the index test	Section 2.4 (LLMs received only image and prompt); Section 2.7 (radiologist readers fully blinded)
**METHODS – Test methods**	13b	Whether clinical information and index test results were available to the assessors of the reference standard	Section 2.1 (reference annotations established independently before this study)
**METHODS – Analysis**	14	Methods for estimating or comparing measures of diagnostic accuracy	Section 2.6 (Wilson CIs, MCC, balanced accuracy, McNemar with Bonferroni, Cochran's Q, Cohen's and Fleiss' kappa)
**METHODS – Analysis**	15	How indeterminate index test or reference standard results were handled	Sections 2.4 and 3 (failures excluded; full failure-case analysis in Section 3.10 and Figure 14)
**METHODS – Analysis**	16	How missing data were handled	Section 3 paragraph 1; Section 3.10 (failure-case analysis)
**METHODS – Analysis**	17	Any analyses of variability in diagnostic accuracy (pre-specified vs exploratory)	Sections 2.6 (subgroup), 2.7–2.12 (reader study, external validation, prompt sensitivity, stability, error taxonomy, failure-case analysis; all pre-specified)
**METHODS – Analysis**	18	Intended sample size and how it was determined	Section 2.1 (5000 for computational feasibility); Section 2.7 (377 stratified-enriched for paired comparison); Section 2.8 (500 sampled, 200 successfully extracted from PadChest)
**RESULTS – Participants**	19	Flow of participants, using a diagram	Figure 1
**RESULTS – Participants**	20	Baseline demographic and clinical characteristics of participants	Section 2.2 (image characteristics); patient-level demographics not in public dataset
**RESULTS – Participants**	21a	Distribution of disease severity in those with the target condition	Section 3 paragraph 2 (per-device abnormal-position prevalence)
**RESULTS – Participants**	21b	Distribution of alternative diagnoses in those without the target condition	Not applicable (device presence/positioning, not disease diagnosis)
**RESULTS – Participants**	22	Time interval and clinical interventions between index test and reference standard	Not applicable (retrospective analysis of the same images)
**RESULTS – Test results**	23	Cross tabulation of index test by reference standard	Supplementary Table S1; Figure 3
**RESULTS – Test results**	24	Estimates of diagnostic accuracy and their precision (95% CIs)	Tables 1, 3, 6, 7 (all with 95% Wilson CIs)
**RESULTS – Test results**	25	Any adverse events	Not applicable (retrospective image analysis, no patient contact)
**DISCUSSION**	26	Study limitations, including sources of bias, statistical uncertainty, and generalizability	Section 4 (limitations subsection): class imbalance for abnormal ETT and NGT; reader-study limitations, including co-author status of readers and enriched-cohort

			design; inference non-standardization across providers; failure-case concentration in multi-device cases; scope limited to general-purpose vs purpose-built/regulator-approved clinical AI; single-source main analysis; API model versioning and reproducibility; HIPAA/GDPR considerations for clinical deployment.
**DISCUSSION**	27	Implications for practice, including intended use and clinical role of the index test	Section 4, final paragraph (adjunct screening role; not autonomous decision-making; not a comparison against specialist clinical AI)
**OTHER INFORMATION**	28	Registration number and registry	Not registered (retrospective study using publicly available data)
**OTHER INFORMATION**	29	Where the full study protocol can be accessed	Sections 2.4–2.12; Supplementary Methods S1; Supplementary Code S1; public datasets on Kaggle (RANZCR CLiP) and BIMCV (PadChest)
**OTHER INFORMATION**	30	Sources of funding and other support; role of funders	Funding section (no external funding received)
**STARD-AI: Model details**	31	Exact AI model identifier (provider, version string, snapshot date)	Section 2.4: gpt-4o-2024-08-06; gemini-3.1-flash-lite-preview (resolved on 15 March 2026); claude-sonnet-4-6 (resolved on 31 March 2026)
**STARD-AI: Model details**	32	Whether the model is general-purpose or purpose-built for the diagnostic task; whether it carries regulatory approval (FDA/CE)	All three are general-purpose multimodal LLMs; none are purpose-built or carry regulatory clearance—explicitly noted in Section 2.4 and Discussion
**STARD-AI: Model details**	33	Training data provenance (if known) and whether overlapping with the test set is possible	Section 2.4: training-data provenance is proprietary to providers; deliberate overlap is not established, but a finite probability of incidental overlap with RANZCR CLiP cannot be excluded
**STARD-AI: Inference parameters**	34	Decoding parameters: temperature, top-p, top-k, max tokens, stop sequences, system prompts	Section 2.4 (defaults documented; max_tokens=500 across all three; system prompt only for GPT-4o)
**STARD-AI: Inference parameters**	35	Whether stochasticity (sampling) is enabled; how it is handled	Section 2.4 (defaults; not zeroed); Section 2.10 + 3.8 (repeat-inference stability characterizes this)
**STARD-AI: Inference parameters**	36	Image preprocessing applied before submission to the model	Section 2.2 (none for RANZCR; native JPEG); Section 2.8 (percentile windowing + JPEG conversion for PadChest 16-bit PNGs)
**STARD-AI: Prompt design**	37	Exact prompt(s) used, including any system message(s)	Section 2.5; Supplementary Methods S1 (V1, V2, V3 in full)
**STARD-AI: Prompt design**	38	Process of prompt selection / engineering and any exploratory subset used	Section 2.5: 200-image exploratory subset, no overlap with the 4813-case analysis

			cohort
**STARD-AI: Prompt design**	39	Prompt sensitivity analysis if multiple prompts could plausibly be used	Section 2.9 + 3.7 (three-variant prompt sensitivity analysis; Figure 11; Table S5)
**STARD-AI: Failure analysis**	40	How model failures (refusals, parsing errors, empty outputs) are handled and whether failures cluster systematically	Section 2.4 (failure modes documented); Section 2.12 + 3.10 (formal failure-case analysis; Figure 14)
**STARD-AI: Stability**	41	Intra-model variability across repeated runs	Section 2.10 + 3.8 (three independent runs; Figure 12; Table S6)
**STARD-AI: Error taxonomy**	42	Systematic enumeration of error types and their model-specific patterns	Section 2.11 + 3.9 (five error categories; Figure 13; Table S7)
**STARD-AI: External validation**	43	External validation on data from independent source(s)	Section 2.8 + 3.6 (PadChest, n=200, presence-detection only; granular position labels not available in PadChest)
**STARD-AI: Comparator**	44	Human-reader or specialist-AI comparator (if any), including blinding and reading conditions	Section 2.7 + 3.5 (blinded paired reader study with two co-author radiologists; viewing conditions, equipment, and expertise documented in Section 2.7)

Items 31–44 represent the subset of STARD-AI 2025 items applicable to a generative-multimodal-LLM diagnostic-accuracy study. Items in the published STARD-AI 2025 that relate to calibration, fairness across protected demographic subgroups, and prospective clinical workflow integration are not applicable to the present retrospective design and are therefore omitted. The mapping additionally covers LLM-specific elements (prompt design, prompt sensitivity, intra-model stability) that are not explicitly enumerated in STARD-AI 2025 but are essential for transparent reporting of generative-AI diagnostic-accuracy studies. Each item is mapped to the manuscript section, figure, table, or supplementary file where it is reported.
