# Supplementary material for: Performance of Multimodal Large Language Models in Detection and Position Assessment of Thoracic Devices on Chest Radiographs
Source: Diagnostics (Basel). 2026 May 23;16(11):1602. doi: 10.3390/diagnostics16111602 (PMC13257059; doi:10.3390/diagnostics16111602)
Supplement: Supplementary file 1 [file diagnostics-16-01602-s001.zip › Table_S4_Reader_Study.pdf]

Supplementary Table S4

Reader Study Additional Metrics

Table S4a. Inter-reader agreement (Cohen's kappa).

Reader 1 = Hamza Eren Güzel; Reader 2 = Cemre Özenbaş. n varies by task because classification is restricted to device-present cases (per agreement of both readers).

Comparison	N	Cohen's Kappa	Percent Agreement	Interpretation
ETT presence	377	0.893	0.950	Almost perfect
ETT abnormal (among present)	223	0.445	0.969	Moderate
NGT presence	377	0.871	0.939	Almost perfect
NGT abnormal (among present)	222	0.290	0.959	Fair
CVC presence	377	0.583	0.931	Moderate
CVC abnormal (among present)	330	0.552	0.867	Moderate
Swan-Ganz presence	377	0.924	0.981	Almost perfect

Table S4b. Paired McNemar comparisons (reader vs. LLM) with Bonferroni correction.

Bonferroni correction applied over 42 comparisons (2 readers × 3 LLMs × 7 device-task pairs). Corrected $p < 0.05$ considered significant. "Better" model determined by accuracy (presence) or MCC (classification).

Task	Device	Comparison	p (raw)	p (corrected)	Better
Presence	ETT	Reader 1 vs GPT	<0.001	<0.001	Reader 1
Presence	ETT	Reader 1 vs Gemini	<0.001	<0.001	Reader 1
Presence	ETT	Reader 1 vs Claude	<0.001	<0.001	Reader 1
Presence	ETT	Reader 2 vs GPT	<0.001	<0.001	Reader 2
Presence	ETT	Reader 2 vs Gemini	<0.001	<0.001	Reader 2
Presence	ETT	Reader 2 vs Claude	<0.001	<0.001	Reader 2
Classification	ETT	Reader 1 vs GPT	<0.001	<0.001	Reader 1
Classification	ETT	Reader 1 vs Gemini	<0.001	<0.001	Reader 1
Classification	ETT	Reader 1 vs Claude	1.000	1.000	Reader 1
Classification	ETT	Reader 2 vs GPT	<0.001	<0.001	Reader 2
Classification	ETT	Reader 2 vs Gemini	<0.001	<0.001	Reader 2
Classification	ETT	Reader 2 vs Claude	0.219	1.000	Reader 2
Presence	NGT	Reader 1 vs GPT	<0.001	<0.001	Reader 1

Presence	NGT	Reader 1 vs Gemini	<0.001	<0.001	Reader 1
Presence	NGT	Reader 1 vs Claude	<0.001	<0.001	Reader 1
Presence	NGT	Reader 2 vs GPT	<0.001	<0.001	Reader 2
Presence	NGT	Reader 2 vs Gemini	<0.001	<0.001	Reader 2
Presence	NGT	Reader 2 vs Claude	<0.001	<0.001	Reader 2
Classification	NGT	Reader 1 vs GPT	<0.001	<0.001	Reader 1
Classification	NGT	Reader 1 vs Gemini	<0.001	<0.001	Reader 1
Classification	NGT	Reader 1 vs Claude	0.289	1.000	Reader 1
Classification	NGT	Reader 2 vs GPT	<0.001	<0.001	Reader 2
Classification	NGT	Reader 2 vs Gemini	<0.001	<0.001	Reader 2
Classification	NGT	Reader 2 vs Claude	0.070	1.000	Reader 2
Presence	CVC	Reader 1 vs GPT	<0.001	<0.001	Reader 1
Presence	CVC	Reader 1 vs Gemini	0.144	1.000	Reader 1
Presence	CVC	Reader 1 vs Claude	<0.001	<0.001	Reader 1
Presence	CVC	Reader 2 vs GPT	<0.001	<0.001	Reader 2
Presence	CVC	Reader 2 vs Gemini	0.801	1.000	Reader 2
Presence	CVC	Reader 2 vs Claude	<0.001	<0.001	Reader 2
Classification	CVC	Reader 1 vs GPT	0.001	0.045	Reader 1
Classification	CVC	Reader 1 vs Gemini	0.007	0.274	Reader 1
Classification	CVC	Reader 1 vs Claude	0.906	1.000	Reader 1
Classification	CVC	Reader 2 vs GPT	<0.001	<0.001	Reader 2
Classification	CVC	Reader 2 vs Gemini	<0.001	<0.001	Reader 2
Classification	CVC	Reader 2 vs Claude	0.010	0.434	Reader 2
Presence	Swan	Reader 1 vs GPT	<0.001	<0.001	Reader 1
Presence	Swan	Reader 1 vs Gemini	<0.001	<0.001	Reader 1
Presence	Swan	Reader 1 vs Claude	<0.001	<0.001	Reader 1
Presence	Swan	Reader 2 vs GPT	<0.001	<0.001	Reader 2
Presence	Swan	Reader 2 vs Gemini	<0.001	<0.001	Reader 2
Presence	Swan	Reader 2 vs Claude	<0.001	<0.001	Reader 2
