# Supplementary material for: Performance of Multimodal Large Language Models in Detection and Position Assessment of Thoracic Devices on Chest Radiographs
Source: Diagnostics (Basel). 2026 May 23;16(11):1602. doi: 10.3390/diagnostics16111602 (PMC13257059; doi:10.3390/diagnostics16111602)
Supplement: Supplementary file 1 [file diagnostics-16-01602-s001.zip › Table_S5_Prompt_Sensitivity.pdf]

Supplementary Table S5

Prompt Sensitivity Analysis: Per-Variant Performance Metrics

Table S5. Per-variant performance metrics (n=103 RANZCR cases).

V1 = baseline (Hamza et al. main study prompt). V2 = chain-of-thought variant. V3 = minimal/concise variant. Full prompt texts in Supplementary Methods S1.

Model	Variant	Device	N pos	Accuracy	Sensitivity	Specificity	MCC	Bal Acc
GPT	V1	ETT	60	0.631	0.533	0.767	0.302	0.650
GPT	V1	NGT	59	0.583	0.424	0.795	0.231	0.610
GPT	V1	CVC	97	0.767	0.814	0.000	-0.114	0.407
GPT	V1	Swan	10	0.825	0.400	0.871	0.221	0.635
GPT	V2	ETT	60	0.650	0.800	0.442	0.260	0.621
GPT	V2	NGT	59	0.670	0.847	0.432	0.311	0.640
GPT	V2	CVC	97	0.718	0.753	0.167	-0.044	0.460
GPT	V2	Swan	10	0.903	0.300	0.968	0.338	0.634
GPT	V3	ETT	60	0.544	0.317	0.860	0.204	0.589
GPT	V3	NGT	59	0.524	0.237	0.909	0.191	0.573
GPT	V3	CVC	97	0.476	0.474	0.500	-0.012	0.487
GPT	V3	Swan	10	0.748	0.600	0.763	0.242	0.682
Gemini	V1	ETT	60	0.777	0.850	0.674	0.536	0.762
Gemini	V1	NGT	59	0.602	0.475	0.773	0.254	0.624
Gemini	V1	CVC	97	0.874	0.928	0.000	-0.067	0.464
Gemini	V1	Swan	10	0.903	0.200	0.978	0.274	0.589
Gemini	V2	ETT	60	0.767	0.783	0.744	0.524	0.764
Gemini	V2	NGT	59	0.631	0.593	0.682	0.272	0.638
Gemini	V2	CVC	97	0.806	0.856	0.000	-0.099	0.428
Gemini	V2	Swan	10	0.864	0.200	0.935	0.150	0.568
Gemini	V3	ETT	60	0.748	0.617	0.930	0.553	0.773
Gemini	V3	NGT	59	0.631	0.508	0.795	0.310	0.652
Gemini	V3	CVC	97	0.825	0.876	0.000	-0.090	0.438
Gemini	V3	Swan	10	0.660	0.700	0.656	0.217	0.678
Claude	V1	ETT	60	0.699	0.583	0.860	0.447	0.722
Claude	V1	NGT	59	0.650	0.610	0.705	0.312	0.657
Claude	V1	CVC	97	0.650	0.670	0.333	0.002	0.502
Claude	V1	Swan	10	0.893	0.000	0.989	-0.032	0.495
Claude	V2	ETT	60	0.699	0.650	0.767	0.412	0.709
Claude	V2	NGT	59	0.621	0.542	0.727	0.270	0.635
Claude	V2	CVC	97	0.796	0.845	0.000	-0.103	0.423
Claude	V2	Swan	10	0.854	0.200	0.925	0.131	0.562
Claude	V3	ETT	58	0.610	0.466	0.810	0.285	0.638
Claude	V3	NGT	56	0.670	0.732	0.591	0.326	0.662
Claude	V3	CVC	94	0.680	0.702	0.333	0.018	0.518
Claude	V3	Swan	10	0.900	0.000	1.000	0.000	0.500

Bal Acc = balanced accuracy. MCC = Matthews correlation coefficient.
