# Supplementary material for: Performance of Multimodal Large Language Models in Detection and Position Assessment of Thoracic Devices on Chest Radiographs
Source: Diagnostics (Basel). 2026 May 23;16(11):1602. doi: 10.3390/diagnostics16111602 (PMC13257059; doi:10.3390/diagnostics16111602)
Supplement: Supplementary file 1 [file diagnostics-16-01602-s001.zip › Table_S6_Stability.pdf]

Supplementary Table S6

Repeat-Inference Stability: Pairwise Cohen's Kappa Across Three Runs

Table S6. Intra-model stability across three independent runs (n=50 cases per run).

Run 1 = original main-study run; Runs 2 and 3 = independent re-runs with the same prompt and inference parameters. κ_xy = Cohen's kappa between Run x and Run y. Mean κ = average of κ_12, κ_13, and κ_23. All-Agree = proportion of cases on which all three runs produced the same classification.

Model	Device	N	κ(R1,R2)	κ(R1,R3)	κ(R2,R3)	Mean κ	All-Agree
GPT	ETT	49	0.295	0.263	0.334	0.297	0.531
GPT	NGT	49	0.169	0.301	0.119	0.197	0.531
GPT	CVC	49	0.375	0.559	0.489	0.474	0.714
GPT	Swan	49	0.104	0.336	0.003	0.148	0.673
Gemini	ETT	50	0.879	0.879	1.000	0.919	0.940
Gemini	NGT	50	0.270	0.214	0.645	0.377	0.600
Gemini	CVC	50	0.811	0.735	0.735	0.760	0.920
Gemini	Swan	50	0.000	0.000	—	0.000	0.980
Claude	ETT	50	0.775	0.775	1.000	0.850	0.900
Claude	NGT	50	0.680	0.719	0.960	0.786	0.840
Claude	CVC	50	0.692	0.780	0.911	0.795	0.860
Claude	Swan	50	0.000	0.000	—	0.000	0.980
