# Supplementary material for: Performance of Multimodal Large Language Models in Detection and Position Assessment of Thoracic Devices on Chest Radiographs
Source: Diagnostics (Basel). 2026 May 23;16(11):1602. doi: 10.3390/diagnostics16111602 (PMC13257059; doi:10.3390/diagnostics16111602)
Supplement: Supplementary file 1 [file diagnostics-16-01602-s001.zip › Table_S7_Error_Taxonomy.pdf]

Supplementary Table S7

Error Taxonomy: Per-Stratum Counts and Rates

Table S7. The error taxonomy on the full 4813-case cohort.

Five error categories systematically enumerated for each model: (1) ETT/NGT confusion; (2) device hallucination on no-device cases (n=3); (3) Swan–Ganz misses; (4) abnormal-position misses; (5) single- vs multi-device per-device accuracy.

Model	Error Type	Count	Eligible	Rate
GPT	ETT-only confused as NGT-only	25	287	0.087
GPT	NGT-only confused as ETT-only	31	262	0.118
Gemini	ETT-only confused as NGT-only	5	287	0.017
Gemini	NGT-only confused as ETT-only	84	262	0.321
Claude	ETT-only confused as NGT-only	23	287	0.080
Claude	NGT-only confused as ETT-only	10	262	0.038
GPT	Hallucinated ETT	1	3	0.333
GPT	Hallucinated NGT	2	3	0.667
GPT	Hallucinated CVC	3	3	1.000
GPT	Hallucinated Swan	1	3	0.333
Gemini	Hallucinated ETT	1	3	0.333
Gemini	Hallucinated NGT	0	3	0.000
Gemini	Hallucinated CVC	3	3	1.000
Gemini	Hallucinated Swan	0	3	0.000
Claude	Hallucinated ETT	0	3	0.000
Claude	Hallucinated NGT	0	3	0.000
Claude	Hallucinated CVC	1	3	0.333
Claude	Hallucinated Swan	0	3	0.000
GPT	Missed Swan-Ganz	91	120	0.758
Gemini	Missed Swan-Ganz	110	120	0.917
Claude	Missed Swan-Ganz	118	120	0.983
GPT	Missed ETT abnormal	9	10	0.900
GPT	Missed NGT abnormal	31	38	0.816
GPT	Missed CVC abnormal	292	347	0.841
Gemini	Missed ETT abnormal	4	10	0.400
Gemini	Missed NGT abnormal	33	38	0.868
Gemini	Missed CVC abnormal	246	347	0.709
Claude	Missed ETT abnormal	10	10	1.000
Claude	Missed NGT abnormal	38	38	1.000
Claude	Missed CVC	340	347	0.980

	abnormal			
GPT	Single-device per-device accuracy	10261	13188	0.778
GPT	Multi-device per-device accuracy	3941	6052	0.651
Gemini	Single-device per-device accuracy	11701	13188	0.887
Gemini	Multi-device per-device accuracy	4767	6052	0.788
Claude	Single-device per-device accuracy	10390	13188	0.788
Claude	Multi-device per-device accuracy	4207	6052	0.695
